# Assessment of the changes in seed yield and nutritional quality of quinoa grown under rainfed Mediterranean environments

**DOI:** 10.3389/fpls.2023.1268014

**Published:** 2023-11-03

**Authors:** Javier Matías, María José Rodríguez, Verónica Cruz, Patricia Calvo, Sara Granado-Rodríguez, Laura Poza-Viejo, Nieves Fernández-García, Enrique Olmos, María Reguera

**Affiliations:** ^1^ Agrarian Research Institute “La Orden-Valdesequera” of Extremadura (CICYTEX), Badajoz, Spain; ^2^ Technological Institute of Food and Agriculture of Extremadura (CICYTEX), Badajoz, Spain; ^3^ Department of Biology, Universidad Autónoma de Madrid, Madrid, Spain; ^4^ Department of Abiotic Stress and Plant Pathology, CEBAS-Consejo Superior de Investigaciones Científicas, Murcia, Spain

**Keywords:** climate change, abiotic stress, rainfed, novel crops, quinoa, nutritional traits, seeds

## Abstract

Climate change is considered a serious threat to agriculture and food security. It is linked to rising temperatures and water shortages, conditions that are expected to worsen in the coming decades. Consequently, the introduction of more drought-tolerant crops is required. Quinoa (*Chenopodium quinoa* Willd.) has received great attention worldwide due to the nutritional properties of its seeds and its tolerance to abiotic stress. In this work, the agronomic performance and seed nutritional quality of three quinoa varieties were studied during two consecutive years (2019-2020) under three water environmental conditions of Southwestern Europe (irrigated conditions, fresh rainfed, and hard rainfed) with the goal of determining the impact of rainfed conditions on this crop performance. High precipitations were recorded during the 2020 growing season resulting in similar grain yield under irrigation and fresh rainfed conditions. However, in 2019, significant yield differences with penalties under water-limiting conditions were found among the evaluated environmental conditions. Furthermore, nutritional and metabolomic differences were observed among seeds harvested from different water environments including the progressive accumulation of glycine betaine accompanied by an increase in saponin and a decrease in iron with water limitation. Generally, water-limiting environments were associated with increased protein contents and decreased yields preserving a high nutritional quality despite particular changes. Overall, this work contributes to gaining further knowledge about how water availability affects quinoa field performance, as it might impact both seed yield and quality. It also can help reevaluate rainfed agriculture, as water deficit can positively impact the nutritional quality of seeds.

## 1 Introduction

Agriculture is facing serious challenges in this century. These include the necessity of feeding an ever-increasing population (which is expected to reach 10 billion by 2050), using more nutritious plant-based food products that will be cultivated in increasingly impoverished soils (Kopittke et al., 2019). This situation is being aggravated by global climate change, which has prompted freshwater shortages that severely affect Mediterranean environments (Zsögön et al., 2022). Therefore, climate change is expected to impact food security and nutrition, resulting in the necessity of undertaking urgent actions to adapt agriculture to new environmental scenarios (Giulia et al., 2020). In line with this, crop diversification through the introduction of stress-resilient crops could greatly contribute to coping with agronomical losses associated with environmental constraints (Rosero et al., 2020).

Currently, the food supply relies on very few crop species which normally require high inputs including irrigation and fertilizers (i.e., maize, rice). Furthermore, global climate predictive models expect more frequent and intense high-temperature waves and erratic and lower rainfalls with increasing series of dry years for the following decades which, consequently, will compromise crop productivity worldwide (Farooq et al., 2011). In fact, the global temperature is expected to increase between 1.4°C and 5.8°C, on average, by the next century, prompting a significant decrease in freshwater resources and, therefore, crop yields (Misra, 2014).

The Mediterranean region is especially vulnerable to the effects of climate change which will impact severely agriculture (Hoegh-Guldberg et al., 2018). This is linked to limiting water availability, especially critical for summer crops, urging to find alternative crops with lower water requirements and higher drought tolerance (Giulia et al., 2020).

Quinoa (*Chenopodium quinoa* Willd.) is a C3 plant that belongs to the Amaranthaceae family, originating from the Andean region and consumed in this region for 7,000 years. Nonetheless, it was not until the middle of the last century when the interest in this crop was globally expanded and its potential was rediscovered (Bazile et al., 2016). This has been in part due to the high nutritional value of its seeds [quinoa seeds are gluten-free and possess a prominent protein content (9%–23%) with a balanced amino acid profile, high-quality fat, and antioxidants (Angeli et al., 2020; Rodríguez Gómez et al., 2021)] but also to the stress tolerance to different environmental factors such as salinity or drought (Killi and Haworth, 2017; Saddiq et al., 2021). The genetic diversity of quinoa confers this plant the potential of growing under a wide range of environments, including unfavorable soil and climatic conditions (Khaled et al., 2021), and, consequently, is a well-recognized climate-resilient plant that constitutes an interesting alternative to traditional crops for new climate change scenarios (Bazile et al., 2016; Hinojosa et al., 2018; Ahmadi et al., 2019). Thus, quinoa has the potential to contribute to minimizing food insecurity worldwide and has the capacity to grow under rainfed conditions in arid and semiarid regions, where water is scarce (Bhargava et al., 2006), including the Mediterranean region where there is an increasing interest in its cultivation (Chaudhary et al., 2023). In line with this, the tolerance of quinoa to water stress has been associated with water-saving strategies that the plant triggers under drought, including protectant mechanisms and an inherent low osmotic potential that involve the synthesis of antioxidant metabolites or organic solutes, like proline, carotenoids, and total soluble sugars, that help to maintain cell turgor pressure, together with different adaptive morphological responses, such as a reduced leaf area (Jacobsen et al., 2009; Hinojosa et al., 2018; Khaled et al., 2021; Saddiq et al., 2021; Maestro-Gaitán et al., 2022; Hamoud et al., 2023).

Although photoperiod was considered the main obstacle for introducing quinoa cultivation in Europe (Bertero, 2001), there are currently several quinoa varieties well adapted to the European photoperiod conditions, being the next crucial aspect to improve its performance under different water environmental conditions (WECs), including those dedicated to rainfed agriculture, which represents large areas of farmland within the European Mediterranean region. Indeed, within Southern Europe, rainfed lands are the vast majority of the arable land. Only in Spain, rainfed farming covers over 84% of the agricultural land (Oduor et al., 2023).

Thus, considering that very few studies have assessed the quinoa performance (in terms of yield and nutritional quality) under drought stress conditions in the field (Saddiq et al., 2021) and aiming at evaluating the potential of cultivating quinoa under rainfed in the Mediterranean Basin of Southwestern Europe, this study has examined the effects of irrigated and rainfed field conditions on yield components and seed nutritional quality-related traits of three quinoa varieties.

## 2 Materials and methods

Three quinoa varieties adapted to European conditions, Pasto and Marisma [kindly provided by the company Algosur S.A. (Lebrija, Spain)] and Titicaca [kindly supplied by the company Quinoa Quality (Copenhagen, Denmark)], were evaluated for two consecutive years (2019, 2020) in three different field water environmental conditions in Extremadura (Spain): irrigated (I), fresh rainfed (FR), and hard rainfed (HR). The two first conditions, I and FR, were situated at the experimental station “La Orden” that belongs to the Center for Scientific and Technological Research of Extremadura (CICYTEX, Spain), located in the Guadiana Basin (lat. 38°51′10′′N; long. 6°39′10′′W). To study the HR environmental conditions, the field experiment was conducted at the dryland area “Maguilla” (lat. 38°23′29′′N; long. 5°42′28′′W). Data of monthly mean minimum and maximum temperature (Tmin and Tmax) and rainfall were obtained from the weather stations located at the experimental stations of “La Orden” and “Maguilla,” respectively (**Supplementary Figure 1**). The experimental site “Maguilla” presented harsher rainfed conditions than “La Orden” considering the rainfall registered in each site. Thus, in 2019, from the end of the dry summer period (1st of October) to the end of the vegetative period of the crop, which increases the soil water reserve, the rainfall recorded in “La Orden” (279.1 mm) was higher than that registered in “Maguilla” (245.2 mm). Furthermore, during the higher vegetative growth of the crop (March to May), the precipitation in “La Orden” (91.4 mm) was 20% higher than in “Maguilla” (76.4 mm). In 2020, the precipitation in “La Orden” from October 2019 to June 2020 was 393.5 mm, while in “Maguilla,” the precipitation for that period was considerably lower (305.2 mm). From March to May, the rainfall registered in “Maguilla” (151.2 mm) was 26% lower than that recorded in “La Orden” (191.3 mm).

The soil at the experimental plots of “La Orden” (CICYTEX, Spain) presented a sandy loam texture with a pH of 6.9, 0.38% of organic matter, 0.045 dS m^−1^ of electrical conductivity (EC), 0.24% of total N, and 93.4 ppm of P, 57.9 ppm of K, 2,364 ppm of Ca, and 252 ppm of Mg. The soil texture of the field trial at “Maguilla” was clayey, with a pH of 7.6, 0.91% of organic matter, 0.098 dS m^−1^ of electrical conductivity, 0.26% of total N, and 67.8 ppm of P, 404.9 ppm of K, 6,086 ppm of Ca, and 371 ppm of Mg. The crop was fertilized at the rate of 150, 100, and 100 kg ha^−1^ of N, P_2_O_5_, and K_2_O, respectively. According to the soil mineral composition and the rate of fertilization previously described, the levels of macronutrients were not limiting for quinoa growth in any of the studied cases.

The experimental design was a randomized complete block in a split-split plot arrangement with four replications, with years as the main plot, as described in **Supplementary Table 1**. As a subplot, the water environmental conditions (WECs) were set: irrigated (I), fresh rainfed (FR), or hard rainfed (HR) conditions. The variety (Pasto, Marisma, and Titicaca) was set as the sub-subplot.

Each experimental plot consisted of four rows: 10 m long and 0.75 m apart. Sowing was conducted in mid-February, at a dose of 6 kg ha^−1^, using a mechanical plot drill. In the irrigation treatment, water was applied by a drip irrigation system to maintain the soil under non-limiting water conditions. Plants were harvested at physiological maturity. In 2019, harvesting was conducted in June for FR (11th) and HR (19th) conditions and in July for I (10th) conditions. In 2020, harvesting was conducted at the end of July for HR (23rd) conditions and at the beginning of August (8th) for FR and I conditions. The sampling area was 3 m^2^ per elemental plot and was performed manually. Then, the seeds were separated using a stationary thresher (Wintersteiger LD 352, Ried, Austria).

### 2.1 Seed nutritional quality parameters

#### 2.1.1 Proximate composition analysis

Proximate analysis [humidity, crude fat, protein, total dietary fiber (TDF), carbohydrate, and ash contents], mineral composition, fatty acid composition, sugars, and saponin content were analyzed following the methodology described by Rodríguez Gómez et al. (2021).

Harvest index (HI) was calculated as in Matías et al. (2021), being the ratio between the seed yield (G) and the total biomass [G + aboveground biomass (AGB)].

#### 2.1.2 NMR analysis for metabolite quantification

Samples were homogenized using a Retsch MM400 (Retsch GmbH, Haan, Germany) and stored at room temperature. Metabolites were extracted from 50 mg of homogenized quinoa seeds with 1 mL of methanol (CH_3_OH:H_2_O) (1:1). The mixture was vortexed followed by 3 min of intermittent sonication (1 min of sonication and a 1 min break, 3 times) and left at 4°C for 30 min. After 20 min of centrifugation (11,000×*g*) at 4°C, the methanol supernatant phase was transferred into 5 mL centrifuge tubes and dried in a speed vacuum (Concentrator plus/Vacufuge plus 5305, Eppendorf AG, Hamburg, Germany). Dried samples were resuspended in 800 µL of 100 mM of potassium phosphate monobasic buffer (KH_2_PO_4_) pH = 6 in 100% deuterium oxide (D_2_O) for NMR analysis, with 0.58 mM of internal standard TSP-d4 (deuterated trimethylsilylpropionic acid sodium salt) (Van der Sar et al., 2013). ^1^H-NMR spectra were recorded at 300.1 ± 0.1 K without rotation and with 4 test scans before the 32 scans performed for the experiment in a Bruker AVIII HD500NMR spectrometer (500.13 MHz for ^1^H) equipped with a 5-mm BroadBand Observe cryogenic probe (BioSpin, Rheinstetten, Germany). The acquisition parameters were as follows: the size of the free induction decays (FIDs) = 64K, spectral band = 12.4345 ppm, receiver gain = 28.5, acquisition time = 2.18 s, relaxation delay = 2 s, and line broadening = 0.50 Hz. A standard one-dimensional pulse sequence NOESY (Bruker 1D, noesypr1d) was used to obtain metabolic profiles of plant extracts through the pulse sequence of presaturation with water suppression using the irradiation of the water frequency during the recycling and mixing times. During sample processing and for each spectrum separately, a reduction of noise was produced, based on the spectral deconvolution of multilevel signal, a baseline correction was performed, and an interpolation technique of the areas of the signal was utilized to complete the process. This provided a “fingerprint” of the sample, a general view of the metabolites with a higher representation, expressing the chemical shifts (*δ*) in ppm (**Supplementary Table 6**). The acquisition spectrum showed the signals and records as frequency vs. intensity. The ^1^H-NMR spectra were processed, and the metabolomics analysis was obtained using the Chenomx NMR Suite software, version 8.3 (Chenomx, Edmonton, Canada). Samples were processed by calibration with the reference peak of the internal standard (TSP-d4). Absolute levels of metabolites were calculated, as µmol per gram of seed extracts, from the least overlapping NMR signals of metabolites and TSP with known concentration, assuming little intersample variations of spin-lattice relaxation time for the same protons.

### 2.2 Statistical analysis

A three-way analysis of variance (ANOVA) was used to evaluate the effect of the year, the genotype, the environmental conditions, and their interactions on the agronomic performance and nutritional characteristics of quinoa varieties. The year was treated as a fixed factor. When the *F* ratio was significant (*p* < 0.05), the *post-hoc* Tukey’s test was performed and used to compare means. Correlations among agronomical and nutritional parameters were evaluated with a Pearson’s correlation coefficient test, and principal component analysis (PCA) was performed with the agronomical and nutritional traits to reduce the number of variables. Analyses were performed using the SPSS Statistics 26.0 (IBM SPSS Inc., New York, NY, USA) analytical software. Correlograms were plotted using the corrplot package (v0.92) (Wei and Simko, 2021) running under R (v4.0.2) (R Core Team, 2021) in RStudio (1.4.1717) (RStudio Team, 2021).

## 3 Results and discussion

### 3.1 Yield parameters

As observed in **Table 1**, the year showed a significant influence on the seed yield. The seed yield was considerably higher (47.6%) in 2020 (1,816 kg ha^−1^) than in 2019 (1,230 kg ha^−1^). When considering the effect of the water environmental conditions, it can be highlighted that the average seed yield was dramatically lower under HR (1,014 kg ha^−1^) compared with I (2,064 kg ha^−1^), reaching an intermediate result under FR conditions (1,595 kg ha^−1^), with significant differences among treatments. Furthermore, the Y × WEC interaction was significant for all yield parameters tested (**Table 1**). However, when comparing treatments within years (**Supplementary Table 2**), it was observed that in 2019 the seed yield was similar under both rainfed treatments (FR: 932 kg ha^−1^ and HR: 842 kg ha^−1^), which was lower than the yield achieved under I (2,000 kg ha^−1^). In contrast, in 2020, the seed yield of FR (2,259 kg ha^−1^) was superior to HR (1,247 kg ha^−1^) and similar to I (2,009 kg ha^−1^). In this work, rainfall differed between years, impacting yield parameters, which were also dependent on the other factors analyzed, including the WEC, the variety, and their interactions. The relatively low rainfall of 2019 was insufficient to satisfy the water crop requirements causing plant water stress, resulting in yield penalties in the non-irrigated plots (**Supplementary Table 2**), as previously observed in former quinoa field studies (Maliro et al., 2017). However, the larger amount of precipitation in 2020, especially in the La Orden experimental station, together with the inherent drought tolerance of quinoa (Khaled et al., 2021) prevented plants from suffering water stress, achieving similar yields in FR than in I. However, in Maguilla (HR), although the rainfall was larger in 2020 than in 2019 (still lower than in La Orden), water stress was strong enough to cause yield penalties in this area, as it occurred in 2019. Furthermore, the lower relative humidity (RH) found in the Maguilla experimental station (HR) probably contributed to the worsening of the water soil stress (Zhang et al., 2021). Interestingly, despite the huge genetic diversity of quinoa that is translated into fluctuations in plant productivity (Ahmadi et al., 2019; Matías et al., 2021), the variety did not influence seed yield (**Table 1**), which can be partially explained because the varieties used in this study are relatively well adapted to the European conditions, showing a similar response among genotypes to limiting water conditions, although not under heat stress (Matías et al., 2021).

**Table 1:** Mean and significance of the seed yield (kg ha^−1^), harvest index (HI), and 1,000-seed weight (g) of three quinoa varieties (Pasto, Marisma, and Titicaca) grown under three water environmental conditions (I, FR, and HR) during two consecutive years (2019 and 2020).

Treatment	Seed yield (kg ha^−1^)	HI	1,000-seed weight (g)
Significance			
Year (Y)	**	*	**n.s.**
Water environmental conditions (WECs)	***	***	***
Variety (V)	n.s.	*	*
Y × WEC	***	***	***
Y × V	n.s.	n.s.	n.s.
WEC × V	n.s.	n.s.	n.s.
Y × WEC x V	n.s.	n.s.	n.s.
Means			
Year (Y)			
2019	1,230 b	0.39 b	2.30
2020	1,816 a	0.42 a	2.38
HSD	209	0.02	0.24
Water environmental conditions (WECs)			
I	2,064 a	0.43 a	2.53 a
FR	1,595 b	0.44 a	2.47 a
HR	1,014 c	0.35 b	2.00 b
HSD	235	0.03	0.13
Variety (V)			
Pasto	1,594	0.39 b	2.21 b
Marisma	1,548	0.40 b	2.25 b
Titicaca	1,532	0.43 a	2.57 a
HSD	320	0.06	0.23

Different lowercase letters within the same column indicate significant differences at p < 0.05 according to Tukey’s test. HSD: critical value for comparison. n.s., not significant; significant at *p < 0.05, **p < 0.01, and *** p < 0.001.

I, irrigated; FR, fresh rainfed; HR, hard rainfed; P, Pasto; M, Marisma; T, Titicaca.

On the other hand, the HI was significantly higher in 2020 (0.42) than in 2019 (0.39) (**Table 1**), which can be explained because of the lower rainfalls of 2019. Furthermore, the HI resulted remarkably lower under HR (0.35) than under I (0.43) and FR (0.44). Nonetheless, when the Y × WEC interaction was analyzed (**Supplementary Table 2**), it was observed that the HI was quite similar under rainfed and irrigated conditions ranging from 0.41 to 0.44, except in 2019 under HR, in which the HI decreased sharply (0.28) due to the more severe water stress conditions. When drought stress occurs at seed filling stage in quinoa, seed yield may decrease dramatically (Gámez et al., 2019; Hinojosa et al., 2019; Saddiq et al., 2021; Maestro-Gaitán et al., 2022). The HI was also dependent on the variety, being higher in Titicaca (0.43) than in Pasto (0.39) and Marisma (0.40), which is probably related to the tolerance of Titicaca variety to arid conditions, as supported by Alvar-Beltrán et al. (2020). In line with this, when evaluating the WEC × V interaction, Titicaca showed an HI decrease between the HR and the I conditions (13%), considerably lower than the decrease observed for Pasto (24%) and Marisma (21%). The average HI achieved in this work (0.41) was slightly lower than the one achieved in a previous work performed under irrigated conditions (0.51) (Matías et al., 2021), but in line with the results previously reported for quinoa by several authors (Curti et al., 2014; Maliro et al., 2017). The lower HI of quinoa compared with those of traditional crops, close to 0.6 (Hay, 1995), reflects that quinoa is not still a fully domesticated crop, as pointed out by other authors (López-Marqués et al., 2020), and implies that it still has the potential to increase seed yield significantly.

The 1,000-seed weight was not influenced by the year (**Table 1**). The average 1,000-seed weight showed a similar trend to the HI, being lower under HR (2.00 g) than under I (2.53 g) and FR (2.47 g), similar to the results previously observed in quinoa (Khatab et al., 2022). Nevertheless, when considering the Y × WEC interaction, the impact of rainfed conditions on the 1,000-seed weight was only significant in 2019 under HR. Taking into account that these quinoa varieties have previously shown a drought-scape strategy, consisting of shortening of the overall life cycle and specific stages like the seed filling period (Maestro-Gaitán et al., 2022), this decrease in seed weight could also be explained due to the more stressful water conditions in 2019 under HR, which would probably have shortened the duration of the seed filling stage, reducing the seed loading period, which is determinant of the final seed weight (Sehgal et al., 2018; Maestro-Gaitán et al., 2022). The variety (genotypic factor) also affected the 1,000-seed weight. A significantly larger HI was determined in Titicaca (2.57 g) than in Pasto (2.21 g) or Marisma (2.25 g). On the other hand, both the highest and the lowest 1,000-seed weight were achieved in 2019 (2.82 g for I and 1.81 for HR).

### 3.2 Nutritional characterization of quinoa seeds

Aiming at evaluating the impact of rainfed conditions on the nutritional quality of quinoa, a proximate composition analysis was performed on seeds obtained from the three varieties. The seed nutritional composition of quinoa is a crucial aspect when evaluating this crop’s performance due to the great potential of this plant to contribute to food security worldwide (Ruiz et al., 2014). In this work, it has been observed that nutritional properties can vary depending on the environmental conditions and the genotype × environment interaction, as has been pointed out by different authors (Curti et al., 2014; Hinojosa et al., 2018; Reguera et al., 2018; Matías et al., 2021),

Significant changes between years were found in the humidity, fiber, and CH contents, energy value, and mineral composition (**Table 2**; **Supplementary Table 3**), which could be mainly explained by the different weather conditions. The humidity was higher in 2020 (10.1%) compared with 2019 (9.6%), as well as the fiber content (11.0% in 2020; 8.5% in 2019). However, the average content of CH and energy value were higher in 2019 (59.3%, 360.0 kcal 100 g^−1^) than in 2020 (55.7%, 350.3 kcal 100 g^−1^). The P, K, and Na contents differed significantly between years. In 2020, the average contents of P (0.41%) and K (1.18%) were higher than in 2019 (P: 0.28%; K: 1.04%), while the Na level was considerably lower in 2020 (55.7 ppm) than in 2019 (118.8 ppm).

**Table 2:** Proximate and mineral composition and saponin content of the seeds of three quinoa varieties (Pasto, Marisma, and Titicaca) grown under three different water environmental conditions (I, FR, and HR) in two consecutive years (2019 and 2020).

Treatment	Humidity (g 100 g^−1^ fw)	Ash (g 100 g^−1^ fw)	Protein (g 100 g^−1^ fw)	Fat (g 100 g^−1^ fw)	Fiber (g 100 g^−1^ fw)	CH (g 100 g^−1^ fw)	Energy (kcal 100 g^−1^ fw)	Saponin (g 100 g^−1^ fw)	P (%)	K (%)	Ca (%)	Mg (%)	Fe (ppm)	Na (ppm)
Significance														
Year (Y)	*	n.s.	n.s.	n.s.	**	**	*	n.s.	*	*	n.s.	n.s.	n.s.	*
Water environmental conditions (WECs)	*	n.s.	**	n.s.	n.s.	n.s.	n.s.	***	**	*	n.s.	**	***	***
Variety (V)	n.s.	**	n.s.	***	***	***	n.s.	n.s.	**	***	n.s.	**	**	n.s.
Y × WEC	*	n.s.	*	n.s.	n.s.	n.s.	n.s.	**	*	*	*	n.s.	n.s.	***
Y × V	n.s.	n.s.	n.s.	***	***	*	***	n.s.	*	n.s.	n.s.	n.s.	n.s.	n.s.
WEC × V	n.s.	n.s.	n.s.	n.s.	***	**	**	n.s.	n.s.	n.s.	n.s.	n.s.	n.s.	n.s.
Y × WEC × V	n.s.	n.s.	n.s.	n.s.	***	**	*	n.s.	n.s.	n.s.	n.s.	n.s.	n.s.	n.s.
Means														
Year (Y)														
2019	9.6 b	3.0	14.1	5.50	8.5 b	59.3 a	360.0 a	1.25	0.28 b	1.04 b	0.12	0.20	52.3	118.8 a
2020	10.1 a	3.3	14.6	5.22	11.0 a	55.7 b	350.3 b	1.33	0.41 a	1.18 a	0.13	0.22	59.4	55.7 b
HSD	0.04	0.4	0.9	0.30	0.8	1.5	4.4	0.26	0.07	0.01	0.04	0.03	24.3	53.7
Water environmental conditions (WECs)														
I	10.1 a	3.1	14.4 b	5.22	9.1	58.5	355.2	0.90 b	0.37 a	1.04 b	0.12	0.22 a	62.6 a	149.4 a
FR	9.9 ab	3.1	13.7 b	5.42	10.3	57.5	354.5	1.48 a	0.37 a	1.17 a	0.14	0.23 a	56.7 b	59.1 b
HR	9.7 b	3.2	15.3 a	5.43	9.9	56.5	355.9	1.49 a	0.30 b	1.12 ab	0.12	0.18 b	48.3 c	53.3 b
HSD	0.3	0.3	0.8	0.27	2.1	2.6	5.5	0.20	0.04	0.10	0.03	0.02	5.6	44.2
Variety (V)														
P	9.9	3.2 a	14.3	5.62 a	10.3 a	56.5 b	354.8	1.30	0.36 a	1.16 a	0.13	0.22 a	61.6 a	93.1
M	9.9	3.3 a	14.4	5.49 a	10.4 a	56.6 b	354.1	1.26	0.36 a	1.19 a	0.13	0.22 a	55.5 ab	87.9
T	9.8	2.9 b	14.4	4.97 b	8.4 b	59.4 a	356.6	1.30	0.32 b	0.97 b	0.12	0.20 b	50.5 b	80.8
HSD	0.03	0.2	1.0	0.32	0.9	1.3	3.2	0.19	0.03	0.07	0.03	0.02	8.1	37.3

Different lowercase letters within the same column indicate significant difference at p < 0.05 according to Tukey’s test. HSD: critical value for comparison. n.s., not significant; significant at *p < 0.05, **p < 0.01, and ***p < 0.001. Proximate composition and saponin content are expressed in fresh weight, while mineral composition is expressed in dry weight.

I, irrigated; FR, fresh rainfed; HR, hard rainfed; P, Pasto; M, Marisma; T, Titicaca.

**Figure 1:**
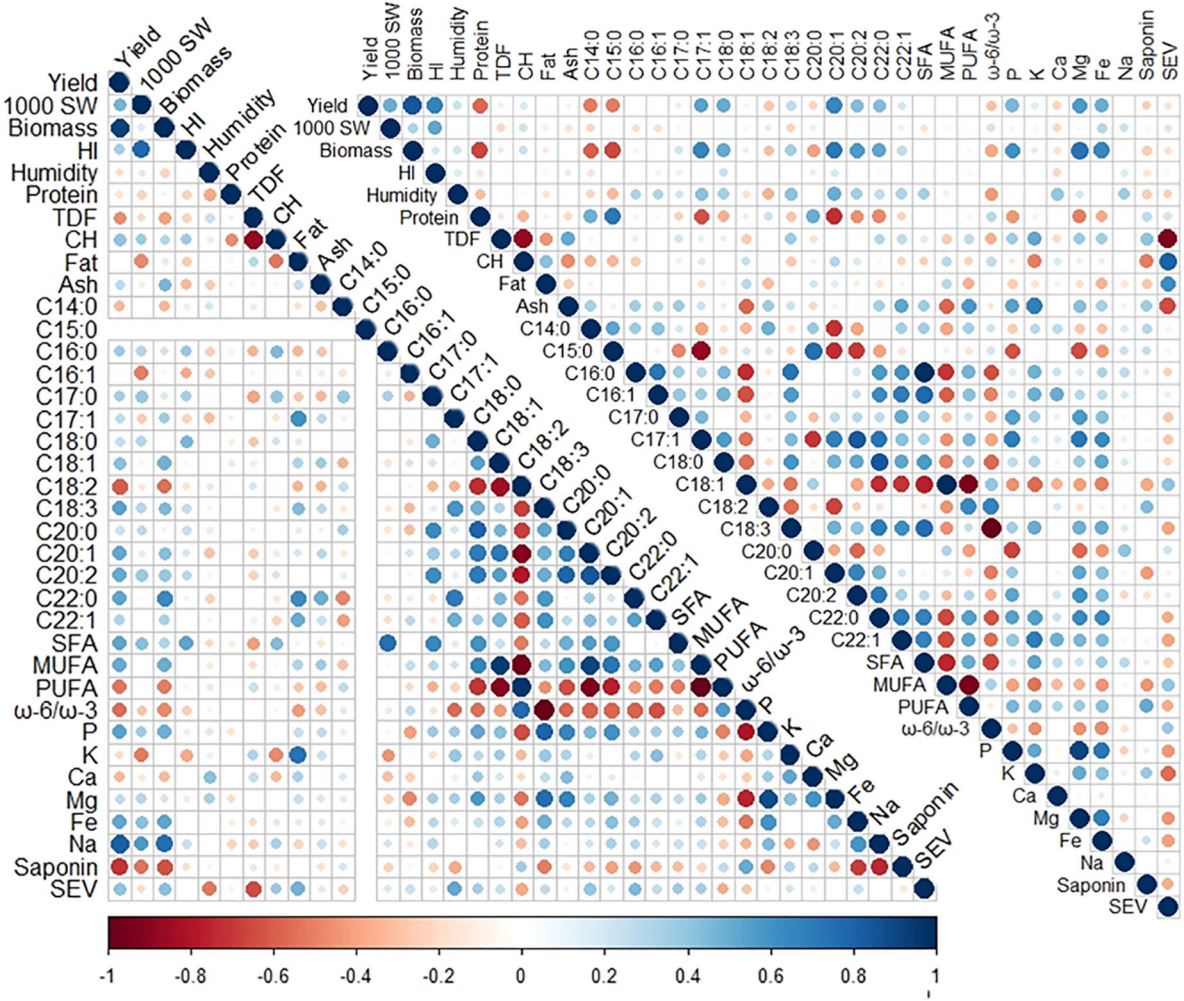
Correlogram of nutritional and agronomical variables measured in 2019 (lower left) and 2020 (upper right). The size and color of the circles indicate Pearson’s correlation coefficient (*r*) between variables, with bigger circles indicating higher correlations and red and blue indicating negative and positive correlations, respectively. One thousand (1,000) SW, 1,000 seeds weight; HI, harvest index; TDF, seed total dietary fiber; CH, carbohydrates; C14:0, seed myristic acid; C15:0, pentadienoic acid; C16:0, palmitic acid; C16:1, palmitoleic acid; C17:0, margaric acid; C17:1, margaroleic acid; C18:0, stearic acid; C18:1, oleic acid; C18:2, linoleic acid; C18:3, linolenic acid; C20:0, arachidic acid; C20:1, gadoleic acid; C20:2, eicosadienoic acid; C22:0, behenic acid; C22:1, erucic acid; SFA, saturated fatty acids; MUFA, monounsaturated fatty acids; PUFA, polyunsaturated fatty acids; ω-6/ω-3, omega-6/omega-3 fatty acid ratio; SEV, seed energy value.

The WECs significantly affected the humidity, protein content, and mineral composition (**Table 2**). Thus, the humidity content achieved the highest average level under I (10.9%) and the lowest content under HR (9.7%). Also, the Y × WEC interaction was significant for humidity although the values were all close to 10%. The protein content achieved a higher average content under HR (15.3 g 100 g^−1^ fw) during the 2 years of experimentation compared with I (14.4 g 100 g^−1^ fw) and FR (13.7 g 100 g^−1^ fw) conditions. This response to water deficit has been also reported in a recent greenhouse experiment (Maestro-Gaitán et al., 2023) that showed that a low irrigation in this crop resulted in a decreased seed yield coupled with an increase in the seed protein content. Furthermore, this trade-off (between protein and seed yield) was significant in 2020 (**Figure 1**) and was consistent with previously published results that showed a negative correlation between these two parameters (Reguera et al., 2018; Granado-Rodríguez et al., 2021). The Y × WEC interaction was significant. In 2019, protein contents were not changing among WECs. However, the protein content under HR in 2020 (16.1 g 100 g^−1^ fw) was significantly higher than those achieved under I (13.9 g 100 g^−1^ fw, 14.2 g 100 g^−1^ fw) and FR (13.9 g 100 g^−1^ fw, 13.6 g 100 g^−1^ fw) for both years (2019, 2020) but similar to that obtained under HR in 2019 (14.6 g 100 g^−1^ fw). Due to the higher rainfall that occurred during the vegetative period of 2020, the crop cycle was approximately a month longer than in 2019, pushing the flowering and seed filling stages to coincide with higher temperatures. Although little is still known about the effect of the high temperatures on the quinoa seed composition (Curti et al., 2020; Matías et al., 2021; Matías et al., 2022), increments in the protein content with high temperatures have been already reported in quinoa (Matías et al., 2021). Quinoa seed is a good source of high-quality plant-based protein, with reported contents ranging from approximately 14% to 18% (De Santis et al., 2016; Ahmadi et al., 2019), in line with the results obtained in this research (14.4%, on average), that were similar to those reported in a previous work performed in the same location under irrigated conditions (Matías et al., 2021). The lipid content was not affected by the WEC contrary to what was observed in Maestro-Gaitán et al. (2023). Thus, no increase in fat content in the seeds was observed under irrigated conditions, unlike that reported for oil crops, such as olive or sunflower (Erdemoglu et al., 2003; Bubola et al., 2022). The WEC influenced significantly the mineral composition of the seeds, as observed by Gonzalez et al. (2012), except Ca. In HR, seeds appeared to have the lowest average levels of P (0.30%), Mg (0.18%), Fe (48.3 ppm), and Na (53.3 ppm). The lower contents of Mg and Fe under HR were probably related to the higher soil water stress (Da Silva et al., 2010), which could result in a lower photosynthetic activity of plants. In this regard, it is well known that Mg and Fe play a role in photosynthesis affecting chloroplast electron transport and chlorophyll synthesis (Hänsch and Mendel, 2009; Farhat et al., 2016). The implication of these two elements in photosyhtesis could explain the positive correlation found between them and the plant biomass and seed yield in the 2020 harvest (**Figure 1**). Also, the levels of P decreased with high soil water stress (HR), which can be explained by the reduced P absorption from the soil under water scarcity, similar to what could occur with Mg and Fe (Bechtaoui et al., 2021). Indeed, drought can lead to nutrient deficiency in plants. In the case of K, the levels were higher under rainfed than under irrigation conditions, which can be related to biochemical mechanisms to maintain turgor pressure by osmotic adjustment, as reported by Saddiq et al. (2021). K plays an important role in many physiological and biochemical processes in plants, such as photosynthesis or plant growth, with the water stress tolerance reduced when K is scarce as it is a key factor controlling stomatal opening (Xu et al., 2020). The Y × WEC interaction significantly affected the contents of P, Ca, and Na, as shown in **Table 2**. Interestingly, the Na content under I in 2019 (**Supplementary Table 3**) was considerably higher (237.5 ppm) compared with the Na contents found in 2020 under rainfed conditions (HR and FR), which is in line with that reported by Gámez et al. (2019). Generally, soil water stress mainly occurred in rainfed treatments of 2019, as explained above. The Y × WEC interaction significantly affected the contents of P, Ca, and Na, as shown in **Table 2**. Interestingly, the Na content under I in 2019 (**Supplementary Table 3**) was considerably higher (237.5 ppm) compared with the Na contents found in 2020 in the other water conditions (HR and FR).

The seed proximate composition was also influenced by the variety, significantly affecting the ash, fat, fiber, CH, and mineral contents. The ash contents in Pasto and Marisma (3.2 and 3.3 g 100 g^−1^ fw, respectively) were higher than in Titicaca (2.9 g 100 g^−1^ fw). The highest fat levels were found in Pasto (5.62%) and Marisma (5.49%) followed by Titicaca (4.97%) (**Table 2**). The average fat content was 5.4%, in line with previously reported values for quinoa. Although quinoa presents a relatively low-fat content (of approximately 5%–7%), the lipid fraction of quinoa is of great interest due to its good quality, mainly due to the presence of polyunsaturated fatty acids (PUFAs). Some authors have even pointed out that quinoa could be used for oil extraction (Curti et al., 2020). The fiber level followed a similar trend as the fat content, achieving the highest content in Pasto (10.3%) and Marisma (10.4%) and the lowest in Titicaca (8.4%). Quinoa seeds also stand out as an important source of dietary fiber (TDF), with contents that normally range between 8% and 14% (Nowak et al., 2016). These values are similar to those found in the same varieties in a greenhouse experiment, and the decrease in the fiber content during the season with lower water availability fits well with the decrease found in the seed fiber content in plants subjected to long-term water stress (Maestro-Gaitán et al., 2022). The values were also slightly lower than those determined at the same location in a previous work (which yielded 16.5% on average) (Matías et al., 2021), but in that case, other varieties were analyzed and only under irrigated conditions. Interestingly, it should be noted that, in both field studies, the highest fiber content was achieved in the year in which the seed filling temperatures were higher. Regarding the CH, the highest content was found in Titicaca (59.4 g 100 g^−1^ fw), while the levels in Pasto and Marisma were similar (56.5 g 100 g^−1^ fw and 56.6 g 100 g^−1^ fw, respectively) (**Table 2**). Furthermore, the mineral composition differed among varieties except for Ca and Na (**Table 2**). Titicaca showed lower levels of P, K, and Mg compared with Pasto and Marisma, as well as a lower Fe content than Pasto. This can be partially explained through the genotypic background of the varieties here analyzed since Titicaca has a different origin than Pasto and Marisma, as previously described.

Saponins are considered undesirable compounds in seeds as they act as antinutrients, although little is known about their molecular functions in plants (Ward, 2000; Otterbach et al., 2021). In this work, the seed saponin content was dependent on the WEC and on the interaction of Y × WEC, as observed in **Table 2** and **Supplementary Table 3**, in line with that reported by Szakiel et al. (2011). Under I conditions, the saponin content achieved the lowest average content (0.90%), while under rainfed conditions, FR and HR, the saponin content achieved higher average values (FR: 1.48%; HR: 1.49%). When evaluating the Y × WEC interaction (**Supplementary Table 3**), in 2019, it was observed that the saponin content was reduced to more than half under I treatment (0.65%) compared with rainfed conditions (FR: 1.56%, HR: 1.46%), in agreement with that observed in other crops (Liao et al., 2017; Chipkar et al., 2022). However, in 2020, no significant differences were found among the WEC treatments. Abiotic and biotic stresses, such as water stress, can trigger the synthesis of secondary metabolites like saponins in plants (Liao et al., 2017; Hamoud et al., 2023). Nonetheless, Pulvento et al. (2012) observed opposite trends with an increase of saponins in quinoa at high levels of irrigation and also when plants were subjected to salinity stress. Interestingly, in our study, saponin contents were similar across locations (Maguilla, La Orden) under the same water management regime (rainfed conditions), which is consistent with that reported by Reguera et al. (2018).

### 3.3 Fatty acid composition of the quinoa seed oil

As previously mentioned, the oil content was similar in both years, with an average of 5.4% (**Table 2**). A total of 15 types of fatty acids were detected and quantified in the seed samples analyzed (**Tables 3**, **4**). By far, PUFAs were the major fatty acids (67.5% of the total), and the ω-6/ω-3 ratio was 10.2, on average. These results are in agreement with those reported by others (Repo-Carrasco et al., 2003; Curti et al., 2020; Matías et al., 2022), confirming the outstanding nutritional quality of quinoa oil. The impact of soil water stress on the quinoa fatty acid composition is unknown to date. Indeed, it remains still unclear in common oil crops like olive and sunflower, in which contrasting results have been published (Erdemoglu et al., 2003; Ayoub et al., 2013; Khoufi et al., 2014; Bubola et al., 2022). In our study, significant variations were observed in the composition of the lipid profile of the quinoa seed oil depending on the WEC, but with relatively fewer quantitative changes that did not drastically affect its nutritional quality.

**Table 3:** Main fatty acid content of the seeds of three quinoa varieties (Pasto, Marisma, and Titicaca) grown under three different environmental conditions (I, FR, and HR) in two consecutive years (2019 and 2020).

Treatment	C16:0	C18:1	C18:2	C18:3	SFA	MUFA	PUFA	ω-6/ω-3
Significance								
Year (Y)	n.s.	**	n.s.	*	*	*	*	*
Water environmental conditions (WECs)	n.s.	n.s.	**	***	n.s.	n.s.	*	***
Variety (V)	*	*	***	***	**	n.s.	n.s.	***
Y × WEC	n.s.	n.s.	n.s.	n.s.	n.s.	n.s.	n.s.	n.s.
Y × V	**	**	n.s.	n.s.	**	**	*	n.s.
WEC × V	n.s.	n.s.	n.s.	n.s.	n.s.	n.s.	n.s.	n.s.
Y × WEC × V	n.s.	**	***	n.s.	n.s.	**	***	n.s.
Means								
Year (Y)								
2019	9.92	20.3 a	60.9	5.7 b	10.7 b	22.5 a	66.8 b	10.8 a
2020	9.64	18.4 b	61.3	6.5 a	11.2 a	20.5 b	68.2 a	9.6 b
HSD	0.37	0.7	0.4	0.5	0.4	0.1	0.8	0.8
Water environmental conditions (WECs)								
I	9.84	19.5	60.2 b	6.4 a	11.0	21.9	66.9 b	9.5 c
FR	9.74	19.4	61.2 a	6.2 a	11.0	21.2	67.8 a	10.0 b
HR	9.75	19.0	61.8 a	5.7 b	10.9	21.4	67.7 ab	11.0 a
HSD	0.17	0.7	0.8	0.3	0.2	0.8	0.7	0.4
Variety (V)								
P	9.77 ab	19.5 a	60.4 b	6.5 a	11.1 a	21.8	67.2	9.5 b
M	9.87 a	18.9 b	61.0 b	6.3 a	11.1 a	21.5	67.7	9.8 b
T	9.69 b	19.5 a	61.8 a	5.6 b	10.8 b	21.2	67.6	11.2 a
HSD	0.16	0.5	0.7	0.3	0.1	0.7	0.7	0.4

Palmitic acid (C16:0); oleic acid (C18:1); linoleic acid (C18:2); linolenic acid (C18:3). SFA (saturated fatty acids): C14:0 + C15:0 + C16:0 + C17:0 + C18:0 + C20:0 + C22:0. MUFA (monounsaturated fatty acids): C16:1 + C17:1 + C18:1 + C20:1 + C22:1. PUFA (polyunsaturated fatty acids): C18:2 + C18:3 + C20:2. ω-3: C18:3. Ω-6: C18:2+ C20:2. Different lowercase letters within the same column indicate significant difference at p < 0.05 according to Tukey’s test. HSD: critical value for comparison. n.s., not significant; significant at *p < 0.05, **p < 0.01, and ***p < 0.001.

I, irrigated; FR, fresh rainfed; HR, hard rainfed; P, Pasto; M, Marisma; T, Titicaca.

**Table 4:** Minor fatty acid contents in seeds harvested from three quinoa varieties (V) grown under three different water environmental conditions (WECs) during two consecutive years (Y).

Treatment	C14:0	C15:0	C16:1	C17:0	C17:1	C18:0	C20:0	C20:1	C20:2	C22:0	C22:1
Significance											
Year (Y)	*	^a^	**	n.s.	*	**	*	n.s.	*	*	*
Water environmental conditions (WECs)	*	***	n.s.	n.s.	***	**	n.s.	*	**	*	n.s.
Variety (V)	n.s.	n.s.	n.s.	n.s.	***	*	n.s.	n.s.	n.s.	***	***
Y × WEC	*	^a^	n.s.	n.s.	***	n.s.	**	n.s.	**	*	n.s.
Y × V	n.s.	^a^	n.s.	n.s.	**	n.s.	n.s.	n.s.	n.s.	n.s.	n.s.
WEC × V	n.s.	n.s.	n.s.	n.s.	***	n.s.	n.s.	n.s.	n.s.	*	n.s.
Y × WEC × V	n.s.	^a^	n.s.	n.s.	***	n.s.	n.s.	n.s.	**	*	n.s.
Means											
Year (Y)											
2019	0.273 a	^a^	0.155 a	0.045	0.082 a	0.375 b	0.267 b	1.53	0.161 b	0.116 b	0.492 b
2020	0.244 b	0.055	0.050 b	0.046	0.066 b	0.531 a	0.306 a	1.46	0.369 a	0.163 a	0.583 a
HSD	0.016		0.031	0.011	0.007	0.049	0.009	0.42	0.123	0.028	0.041
Water environmental conditions (WECs)											
I	0.253 b	0.047 b	0.105	0.048	0.084 a	0.464 a	0.295	1.69 a	0.312 a	0.149 a	0.552
FR	0.258 ab	0.040 b	0.092	0.047	0.089 a	0.482 a	0.286	1.45 ab	0.336 a	0.150 a	0.543
HR	0.266 a	0.079 a	0.110	0.041	0.050 b	0.413 b	0.279	1.34 b	0.146 b	0.119 b	0.517
HSD	0.012	0.001	0.025	0.009	0.013	0.044	0.023	0.32	0.100	0.027	0.035
Variety (V)											
P	0.255	0.059	0.104 ab	0.041	0.076 b	0.471 a	0.294	1.61	0.262	0.156 a	0.555 a
M	0.261	0.054	0.112 a	0.048	0.088 a	0.454 ab	0.286	1.51	0.286	0.161 a	0.571 a
T	0.260	0.052	0.091 b	0.047	0.059 c	0.433 b	0.279	1.36	0.247	0.102 b	0.487 b
HSD	0.012	0.014	0.021	0.008	0.007	0.031	0.027	0.28	0.067	0.017	0.049

I, irrigated; FR, fresh rainfed; HR, hard rainfed; P, Pasto; M, Marisma; T, Titicaca.

a Not detected in 2019. Myristic acid (C14:0); pentadecanoic acid (C15:0) palmitoleic acid (C16:1); margaric acid (C17:0); margaroleic acid (C17:1); stearic acid (C18:0); arachidic acid (C20:0); gadoleic acid (C20:1); eicosadienoic acid (C20:2); behenic acid (C22:0); and erucic acid (C22:1). Different lowercase letters within the same column indicate significant difference at p < 0.05 according to Tukey’s test. HSD: critical value for comparison. n.s., not significant; significant at *p < 0.05, **p < 0.01, and ***p < 0.001.

The major fatty acid found was linoleic acid (C18:2), followed by oleic acid (C18:1), palmitic acid (C16:0), and linolenic acid (C18:3) (**Table 3**). The linolenic acid (C18:3) content was higher in 2020 (5.7%) than in 2019 (6.5%), contrary to oleic acid (C18:1) content that was higher in 2019 (20.3%) than in 2020 (18.4%). The levels of palmitic acid (C16:0) and linoleic acid (C18:2) remained similar in both years, with average values of 9.8% and 61.1%, respectively. Considering the contents of the major fatty acids detected in the quinoa oil fraction, it was found that the WEC affected significantly the linoleic (C18:2) and linolenic (C18:3) acids, but in a different way, showing a negative correlation (**Figure 1**). The average content of C18:2 was significantly lower under I (60.2%) than under FR (61.2%) and HR (61.8%). However, the average C18:3 content was lower under HR (5.7%) than under FR (6.2%) and I (6.4%). The variety was also a factor that influenced all the major fatty acid contents. Marisma and Pasto reached the highest contents of palmitic acid (9.87%, 9.77%) and linolenic acid (6.3%, 6.5%), while the levels of Titicaca for those fatty acids were the lowest (9.69%, 5.6%). Pasto and Titicaca achieved the highest oleic acid content (19.5% in both cases), while Marisma reached the lowest level (18.9%) (**Tables 3**, **4**).

The SFA, MUFA, and PUFA contents and the ω-6 to ω-3 ratio presented a strong influence by the cultivation year (**Table 3**). While the SFA and PUFA contents were significantly lower in 2019 (10.7% and 66.8%, respectively) compared with 2020 (11.2% and 68.2%, respectively), the MUFA content and the ω-6 to ω-3 ratio were larger in 2019 (22.5% and 10.8, respectively) than in 2020 (20.5% and 9.6, respectively). Regarding the comparison among varieties, it was found that the SFA was higher in Pasto (11.1%) and Marisma (11.1%) than in Titicaca (10.8%) and the ω-6 to ω-3 ratio was larger in Titicaca (11.2) than in Pasto (9.5) or Marisma (9.8) (**Table 3**).

Among the minor fatty acid contents, it was observed that the year of cultivation was a significant factor affecting seed composition, except for the heptadecanoic acid (C17:0) and eicosenoic acid (C20:1) (**Table 4**). In the case of myristic acid (C14:0), palmitoleic acid (C16:1), and heptadecenoic acid (C17:1), the contents were higher in 2019, while for the others [pentadecanoic acid (C15:0), stearic acid (C18:0), arachidic acid (C20:0), eicosadienoic acid (C20:2), behenic acid (C22:0), and erucic acid (C22:1)], the contents were higher in 2020. C15:0 was not detected in 2019. The WEC also influenced the minor fatty acid composition. C14:0 showed the lowest content under I conditions, C15:0 achieved the highest content under HR, and the rest of the minor fatty acids showed larger contents under such conditions (I) compared with HR, while no differences were found between I and FR conditions.

### 3.4 Seed metabolomic profile

Metabolite synthesis and accumulation play an essential role in keeping suitable cell osmotic potential in plants facing abiotic stresses (Kumar et al., 2021; Yadav et al., 2021). Quinoa, a facultative halophyte crop, is reported to present a great cellular osmoregulation capacity conferred by osmoprotectants, molecules that contribute to the osmotic potential adjustment of plant tissues to properly maintain enzymatic reactions, cell membrane integrity, and physiological mechanisms along the plant (Delatorre-Herrera et al., 2019). The metabolites found accumulated in quinoa seeds (and determined by ^1^H-NMR) included organic acids, soluble sugars, and free amino acids covering most of the primary metabolism [including glycolysis, tricarboxylic acid (TCA) cycle, and the Shikimate pathway] and some secondary metabolites. The influence of the different factors considered (year, Y; water environmental conditions, WECs; variety, V) on the accumulation of these primary and secondary metabolites was evaluated (**Supplementary Table 7**).

**Figure 2:**
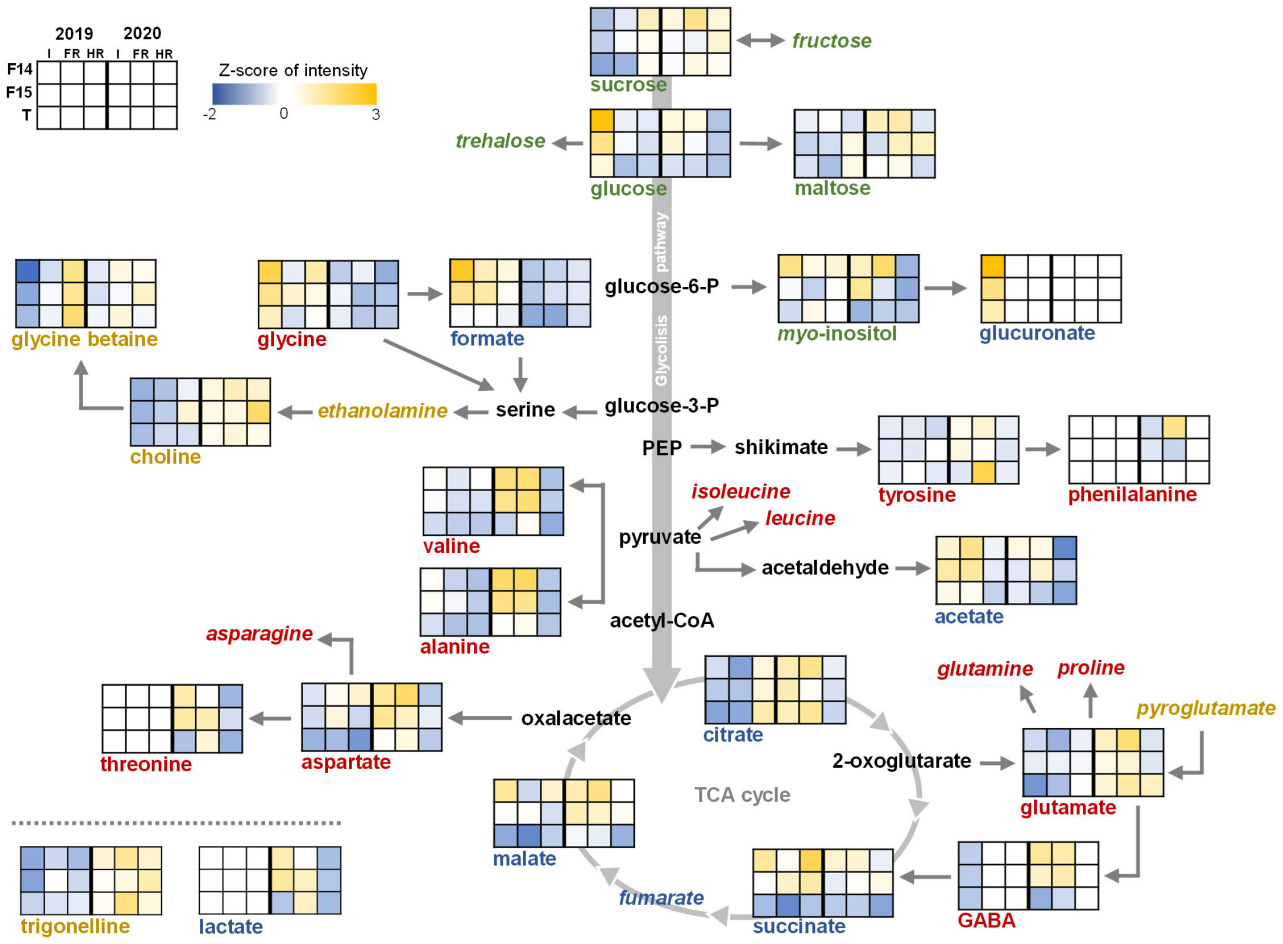
Metabolites quantified in seeds from F14 (Pasto), F15 (Marisma), and T (Titicaca) varieties cultivated under irrigation (I), fresh rainfed (FR), and hard rainfed (HR) conditions during consecutive years 2019 and 2020. In green, sugars; in blue, organic acids; in red, free amino acids; in yellow, other compounds; in black, metabolites not measured. Italics indicate measured but not detected metabolites. Graphs indicate the Z-score from the standardized intensity of each metabolite (three biological replicates).

The accumulation of sucrose in seeds harvested in 2020 showed the highest value among the different sugars analyzed (104.44 µmol/g dw) (**Figure 2**). In addition, sucrose was more abundant under HR conditions (105.06 µmol/g dw) than under I (92.97 µmol/g dw), in any of the varieties analyzed (**Figure 2**; **Supplementary Table 7**). The cultivation year was also a determinant factor of the sucrose accumulation (**Supplementary Table 7**), and the interaction between this factor and the WEC or the variety was also significant (Y × WEC and Y × V). On the other hand, maltose accumulation did not vary considering the factors analyzed, but glucose was differentially accumulated depending on the WEC and the variety considered, showing higher accumulation in I (30.377 µmol/g dw) and in Pasto seeds (23.953 µmol/g dw). As with sucrose, the interaction between the cultivation year and both the WEC and the variety showed a significant influence in the accumulation of this metabolite. The accumulation of *myo*-inositol in quinoa seeds was strongly influenced by the variety, showing an increase in Pasto compared with Marisma and Titicaca (5.8121, 4.7503, and 4.3276 µmol/g dw, respectively). In addition, the interaction between the year and the WEC influenced *myo*-inositol accumulation, as it did in the interaction the WEC and the variety and the interaction of the three factors analyzed.

In the case of the organic acids related to glycolysis and the TCA cycle, the interaction among the three factors analyzed (cultivation year, WEC, and variety) was significant. Furthermore, while citrate, succinate, and malate were present in the quinoa seed samples analyzed, fumarate was not detected. Malate and succinate showed similar accumulation and opposite trends to citrate (**Figure 2**). Moreover, malate and succinate presented a strong influence by the variety, presenting higher accumulation in Pasto and Marisma compared with Titicaca (**Supplementary Table 7**). However, succinate showed no influence by the WEC nor the cultivation year, but malate did by the cultivation year. On the other hand, citrate accumulation was markedly determined by the cultivation year, showing an accumulation of 5.1 µmol/g dw in 2019 and 6.2 µmol/g dw in 2020. Furthermore, when analyzing the metabolomic profile of our quinoa seed samples, the results indicated that, regardless of variety (V), acetate was present in lower levels under HR conditions in comparison with I or FR conditions, regardless of the cultivation year. Acetate has been quite recently described as a metabolite able to mediate drought stress tolerance, regulating the jasmonate signaling pathway through histone acetylation in *Arabidopsis thaliana* (Kim et al., 2017). Furthermore, the role of acetate conferring drought stress tolerance seems to be conserved among different crops, as supplying acetate to the soil before plants are subjected to limited water conditions improves drought tolerance in rice, wheat, and rapeseed (Kim et al., 2017). Still, further research should be performed to explore the roles of this metabolite in water stress response in quinoa. Moreover, the accumulation of acetate was found significantly affected by the cultivation year. Some organic acids were only detected in seeds harvested in a particular year and/or water environmental conditions, such as lactate, which was only found in seeds harvested in 2020, or glucuronate, that was only detected in seeds harvested from irrigated conditions in 2019 (**Figure 2**). Linked to the glycolate pathway, the accumulation of formate was almost doubled in seeds harvested in 2019 than in 2020 (1.2 and 0.8 µmol/g dw, respectively). Furthermore, formate content was influenced by the variety, showing Pasto the largest levels (1.1 µmol/g dw) and Titicaca the lowest (0.8 µmol/g dw).

Nine free amino acids were detected, namely, alanine, aspartate, GABA, glutamate, glycine, phenylalanine, threonine, tyrosine, and valine (**Figure 2**). All of them presented differences depending on the cultivation year, reaching higher values in 2020 than in 2019, except for glycine, whose levels were higher in 2019 (**Supplementary Table 7**). The WEC factor influenced the accumulation of amino acids except for glutamate, threonine, and tyrosine. The variety showed a minor effect, influencing only the accumulation of aspartate, phenylalanine, and valine. The interaction among the cultivation year, the WEC, and the variety was significant for all the amino acids detected except for tyrosine (**Supplementary Table 7**). Alanine and valine presented similar accumulation trends, showing lower levels under HR. In addition, both amino acids were highly accumulated in Pasto and Marisma under I and FR conditions compared with those in Titicaca in 2020 (**Figure 2**). Similar to alanine and valine, the accumulation of aspartate increased under I and FR conditions in 2020 in Pasto and Marisma, while in Titicaca, aspartate levels were stable among WEC and lower among varieties, especially in seeds from 2019.

Within the Shikimate pathway, tyrosine accumulation showed very low and stable levels in all the samples analyzed, only influenced by the cultivation year and the interaction between this factor and the WEC. Phenylalanine was only detected in samples from 2020 in Pasto and Marisma under I and FR conditions (**Figure 2**). Glutamine and proline were not detected in our samples, while glutamate was found accumulated in seeds from 2020, as well as GABA, except for HR conditions (**Supplementary Table 7**). Furthermore, the interaction between the cultivation year and the WEC or the variety showed a strong influence on glutamate accumulation, while for GABA, the interaction between the WEC and the variety or the cultivation year showed the strongest influence.

Other secondary metabolites, such as glycine betaine (GB), choline, and trigonelline, were present in the quinoa seeds analyzed, while ethanolamine, glutathione, and pyroglutamate were not detected (**Figure 2**). Choline was highly accumulated in 2020 and did not show influence by the WEC or the variety (**Supplementary Table 7**). However, the interaction between the cultivation year and the WEC, the cultivation year and the variety, and the interaction among the three factors analyzed affected choline accumulation. Interestingly, WEC was the factor influencing GB content either alone, interacting with the cultivation year, the variety, or all the three factors, reinforcing the idea that rainfed conditions enhanced GB accumulation in Pasto, Marisma, and Titicaca seeds (**Figure 2**; **Supplementary Table 7**). GB was highly accumulated in the seeds harvested from HR conditions and was also more accumulated in FR conditions compared with I conditions, in all the varieties and years analyzed (I = 55.2 µmol/g dw; FR = 67.6 µmol/g dw; HR = 79.7 µmol/g dw). The fact that GB accumulation in quinoa seeds showed a clear gradient trend from its lowest values detected under I conditions to the highest under HR (**Supplementary Table 6**), regardless of V or Y influence, could also be indicative of the role of this metabolite as an osmoprotectant, as previously reported in different plant species such as wheat, barley, or pea subjected to salt and drought stress (Giri, 2011; Annunziata et al., 2019). The Amaranthaceae family includes GB natural accumulator species, and specifically, quinoa GB appeared to be increased in seedlings and mature plants grown under salinity stress conditions contributing to its characteristic halophytic trait (Delatorre-Herrera et al., 2019; Morales, 2009; Ruffino et al., 2009). Therefore, abiotic stressors such as water deficit could trigger GB accumulation playing specific roles in seeds such as protection from detrimental water scarcity damages. Furthermore, trigonelline, another betaine-related osmoprotectant (Yang et al., 2017), was also detected although the levels were only influenced by the cultivation year, showing a greater accumulation in 2020 (2019 = 0.62 µmol/g dw and 2020 = 0.8 µmol/g dw; **Supplementary Table 7**; **Figure 2**). In this case, the cultivation year was the determinant factor influencing trigonelline content, either alone or interacting with the other factors (the cultivation year and/or the WEC).

### 3.5 Correlograms and PCA

Correlations between the agronomical and nutritional parameters measured were analyzed in both years of cultivation. In 2019, there were positive correlations among seed yield, plant biomass, and Na, C18:1, C18:3, C20:1, C22:0 and MUFA contents and negative correlations between those eight variables and the saponin, C18:2, and PUFA contents and the ω-6 to ω-3 ratio. Some significant correlations were also found between the P, Mg, C18:0, C18:3, C20:0, C20:1, and C20:2 contents, and also, those variables negatively correlated with the C18:2 content and the ω-6 to ω-3 ratio. Other correlations were found in 2019 between the seed K and fat contents (*r* = 0.73). The TDF content showed the highest negative correlation with the CH content (*r* = −0.88) and also showed a strong negative correlation with the seed energy value content (*r* = −0.64) but positively correlated with the fat content (*r* = 0.47) (**Figure 1**).

In 2020, a strong correlation between seed yield and biomass was again observed, and these variables correlated with the P, Mg, Fe, C17:1, C18:0, C20:1, C20:2, and C22:0 contents and negatively with the protein, C14:0, and C15:0 contents. Other correlations repeated during the second year were the strong negative correlations found in TDF with the CH content (*r* = −0.87) and with the seed energy value (*r* = −0.92) and the positive correlation between energy value and fat content (*r* = 0.62). In 2020, negative correlations were shown in the fatty acid C18:2 with C18:1 (*r* = −0.57) and C18:3 (*r* = −0.37), and in 2019, a negative correlation between MUFA and PUFA (*r* = −0.95) contents was also found (*r* = −0.85, *r* = −0.67, and *r* = −0.99, respectively) (**Figure 1**). The negative correlation between C18:1 and C18:2 is consistent with previous studies that considered the environmental factor either in quinoa seeds (Rodríguez Gómez et al., 2021; Matías et al., 2022) or in oil crops like sunflower (Erdemoglu et al., 2003). The ash, TDF, C22:1, saponin, P, and K contents positively correlated with each other and showed negative correlations with the fat, CH, C18:1, and MUFA contents and the seed energy value. Among the fatty acids, strong correlations were observed between the C16:0, C17:1, C18:3, C22:0, C22:1, and the total SFA and PUFA contents, which also negatively correlated with the C8:1 and MUFA contents. Other strong positive correlations found in 2020 were those between C17:1 and C20:2 contents (*r* = 0.66) and between the C18:0 and C22:0 contents (*r* = 0.85) (**Figure 1**).

**Figure 3:**
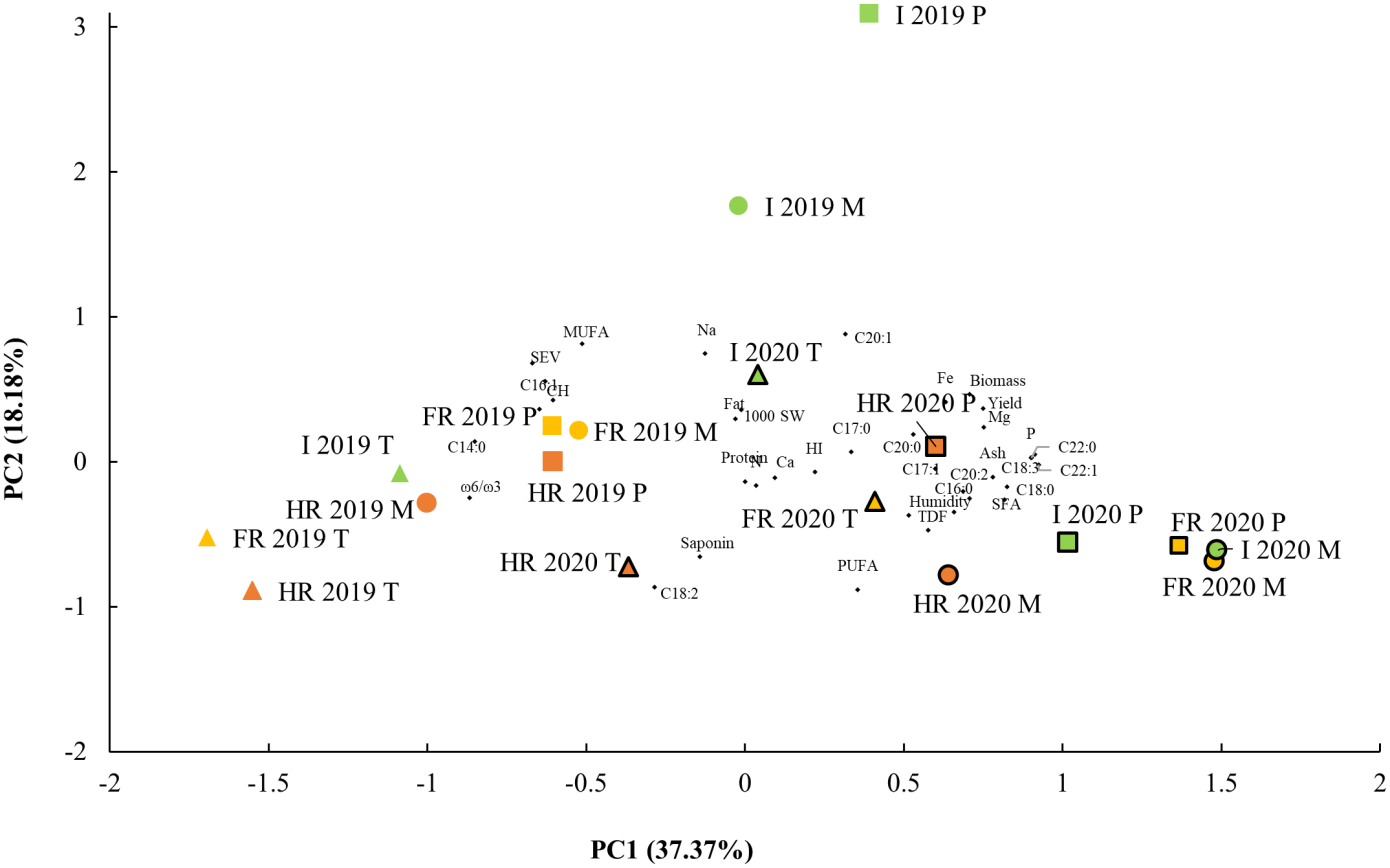
Principal component analysis (PCA). Biplot of main components 1 and 2 agronomical and nutritional traits from three quinoa genotypes: Pasto (P), Marisma (M), and Titicaca (T), grown in different water environmental conditions (WECs): irrigated (I), fresh rainfed (FR), and hard rainfed (HR), during two consecutive years, 2019 and 2020. One thousand (1,000) SW, 1,000 seeds weight; HI, harvest index; TDF, seed total dietary fiber; CH, carbohydrates; C14:0, seed myristic acid; C15:0, pentadienoic acid, C16:0, palmitic acid; C16:1, palmitoleic acid; C17:0, margaric acid; C17:1, margaroleic acid; C18:0, stearic acid; C18:1, oleic acid; C18:2, linoleic acid; C18:3, linolenic acid; C20:0, arachidic acid; C20:1, gadoleic acid; C20:2, eicosadienoic acid; C22:0, behenic acid; C22:1, erucic acid; SFA, saturated fatty acids; MUFA, monounsaturated fatty acids; PUFA, polyunsaturated fatty acids; w-6/w-3, omega-6/omega-3 fatty acid ratio; SEV, seed energy value.

Aiming at reducing the variables to analyze, a PCA was performed, including every agronomic and nutritional variable. Five principal components (PCs) were obtained, which explained a total variance of 83.15% (**Figure 3**). The PC1 contributed to 37.37% of the variance and comprised variables like seed yield, plant biomass, humidity, ash, TDF, P, K, Mg, Fe, C16:0, C17:1, C18:0, C18:3, C20:0, C20:2, C22:0, C22:1, and SFA contents, and the variables that contributed negatively to PC1 were the seed CH, C14:0, C16:1, C18:1, and MUFA contents, and the ω-6 to ω-3 ratio and seed energy value. Samples from 2020 showed higher PC1 values than the 2019 samples, and in both sowing years, Marisma and Pasto varieties showed higher PC1 values than Titicaca (**Figure 3**), consistent with Titicaca’s lower contents observed in seed ash, TDF, P, K, Mg, Fe, C18:3, and SFA (including C16:0, C18:0, and C22:0) and higher contents of carbohydrates and C18:1 (**Tables 2** **–** **4**).

The PC2 explained 18.18%. The variables that positively contributed to the PC2 were the plant biomass, the seed energy value, and the CH, Fe, Na, C18:1, C20:1, and MUFA contents, and those that negatively contributed to the PC2 were the TDF, saponin, C18:2, and PUFA contents. There were higher PC2 values in 2019 in general, but the highest values were found in I in Marisma and Pasto and the lowest value in HR in 2019 in Titicaca (**Figure 3**), so there is a clear interaction between the year of sowing, the WEC, and the genotype factors influencing PC2.

When plotting the PC1 and PC2 values, three main groups were differentiated (**Figure 3**). The first group showed higher PC1 and low PC2 values and comprised the 2020 samples in FR in all varieties and in HR and I in Pasto and Marisma, while the second group, with lower PC1 values, comprised the 2019 HR and FR samples and the Titicaca samples from I in 2019 and HR in 2020 (**Figure 3**). A third group showed intermediate PC1 values but higher PC2 values, suggesting higher CH, Fe, Na, and MUFA contents and lower saponin, CH, and C18:2 contents. The main groups being clearly separated by the year of cultivation are consistent with the significant effect of this factor observed in most of the measured parameters (**Tables 1** **–** **4**).

The PC3 contributed to 11% of the variance and was constituted by the 1,000-seed weight, harvest index, and C17:0 and negatively by the fat and K contents. The highest PC3 values were found in FR and I Titicaca samples in 2019, while the lowest values were those from Pasto and Marisma in the HR in 2019. The PC4 explained 9.26% of the variance and included the C17:1 and C20:2 contents and negatively the protein and C20:0 contents. The 2020 FR samples showed the highest PC4 values and the 2020 HR samples showed the lowest. Lastly, 7.35% of the variance was explained by the PC5, which consisted of the fat, K, Ca, and C20:0 contents.

## 4 Conclusions

In this study, the seed yield ranged from 680 kg ha^−1^ obtained under hard rainfed (HR) to 2,400 kg ha^−1^ achieved under irrigation conditions (I), demonstrating that water stress impacts significantly seed yield in quinoa. The nutritional quality of its seeds was not drastically altered under the different water environmental conditions evaluated (irrigated or rainfed conditions). For instance, the total oil content of quinoa seeds was similar under irrigated and rainfed conditions. However, in the HR, the linoleic content was higher and the linolenic content was lower; therefore, the ω-6/ω-3 was higher but kept healthy ratios. Furthermore, higher protein and saponins were found under more severe rainfed conditions (HR). When comparing among varieties, Titicaca showed larger differences compared with the other varieties used, especially in the lipid content and composition. Therefore, overall, the comparative analyses here performed, integrating agronomical, nutritional, and metabolomic data, reveal significant seed yield penalties and nutritional changes associated with water limitation which highlights the impact of environmental conditions on food security and quality that should be considered under new climate scenarios.

## Data availability statement

The raw data supporting the conclusions of this article will be made available by the authors, without undue reservation.

## Author contributions

JM: Conceptualization, Data curation, Formal Analysis, Funding acquisition, Investigation, Methodology, Project administration, Resources, Supervision, Validation, Visualization, Writing – original draft, Writing – review & editing. MR: Conceptualization, Data curation, Formal Analysis, Investigation, Methodology, Writing – original draft. VC: Data curation, Investigation, Methodology, Writing – original draft. PC: Data curation, Investigation, Methodology, Writing – original draft. SG-R: Investigation, Writing – original draft, Software. LP-V: Investigation, Software, Writing – original draft, Formal Analysis. NF-G: Investigation, Writing – original draft. EO: Investigation, Writing – original draft. MR: Investigation, Writing – original draft, Conceptualization, Data curation, Formal Analysis, Funding acquisition, Methodology, Project administration, Resources, Supervision, Validation, Visualization.
