# Supplementary figures and images for: Assessment of the changes in seed yield and nutritional quality of quinoa grown under rainfed Mediterranean environments
Source: Front Plant Sci. 2023 Nov 3;14:1268014. doi: 10.3389/fpls.2023.1268014 (PMC10662129; doi:10.3389/fpls.2023.1268014)

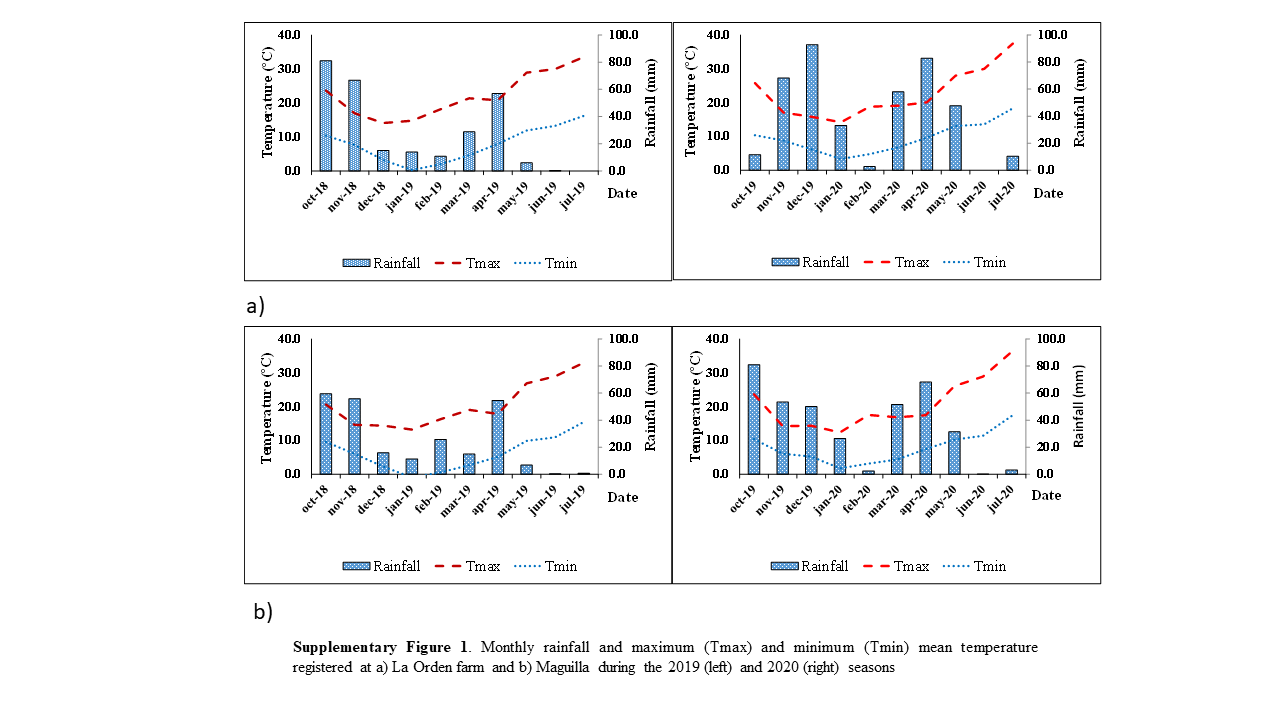

Supplement: Supplementary file 1 [file Image_1.tif]
