# Supplementary material for: Assessment of the changes in seed yield and nutritional quality of quinoa grown under rainfed Mediterranean environments
Source: Front Plant Sci. 2023 Nov 3;14:1268014. doi: 10.3389/fpls.2023.1268014 (PMC10662129; doi:10.3389/fpls.2023.1268014)
Supplement: Supplementary file 2 [file Table_1.docx]

**Table S1:** Experimental design.

| YEAR | Environmental conditions | vARIETY | irrigation application |
| --- | --- | --- | --- |
| 2019 | Irrigation (I) | Pasto (F14) | Yes |
|  |  | Marisma (F15) |  |
|  |  | Titicaca (T) |  |
|  | Fresh rainfed (FR) | Pasto (F14) | No |
|  |  | Marisma (F15) |  |
|  |  | Titicaca (T) |  |
|  | Hard rainfed (HR) | Pasto (F14) | No |
|  |  | Marisma (F15) |  |
|  |  | Titicaca (T) |  |
| 2020 | Irrigation (I) | Pasto (F14) | Yes |
|  |  | Marisma (F15) |  |
|  |  | Titicaca (T) |  |
|  | Fresh rainfed (FR) | Pasto (F14) | No |
|  |  | Marisma (F15) |  |
|  |  | Titicaca (T) |  |
|  | Hard rainfed (HR) | Pasto (F14) | No |
|  |  | Marisma (F15) |  |
|  |  | Titicaca (T) |  |
