# Supplementary material for: Assessment of the changes in seed yield and nutritional quality of quinoa grown under rainfed Mediterranean environments
Source: Front Plant Sci. 2023 Nov 3;14:1268014. doi: 10.3389/fpls.2023.1268014 (PMC10662129; doi:10.3389/fpls.2023.1268014)
Supplement: Supplementary file 3 [file Table_2.docx]

**Table S2**. Seed yield (kg ha^-1^), harvest index (HI), and 1000-seed weight (g) of three quinoa varieties (V) grown under three water environmental conditions (WEC) during two consecutive years (Y) according to treatments interactions.

| **Interaction** | **Seed yield (kg ha^-1^)** | **HI** | **1000-seed weight (g)** |  |
| --- | --- | --- | --- | --- |
| **Y x WEC** |  |  |  |  |
| 2019 x I | 2000 a | 0.43 a | 2.82 a |  |
| 2019 x FR | 932 b | 0.44 a | 2.46 ab |  |
| 2019 x HR | 842 b | 0.28 b | 1.81 c |  |
| 2020 x I | 2009 a | 0.44 a | 2.28 b |  |
| 2020 x FR | 2259 a | 0.42 a | 2.43 ab |  |
| 2020 x HR | 1247 b | 0.41 a | 2.26 bc |  |
| **Y x V** |  |  |  |  |
| 2019 x P | 1389 | 0.35 | 2.24 |  |
| 2019 x M | 1325 | 0.37 | 2.21 |  |
| 2019 x T | 1061 | 0.44 | 2.65 |  |
| 2020 x P | 1869 | 0.42 | 2.23 |  |
| 2020 x M | 1873 | 0.43 | 2.32 |  |
| 2020 x T | 1773 | 0.42 | 2.42 |  |
| **WEC x V** |  |  |  |  |
| I x P | 2189 | 0.42 | 2.47 |  |
| I x M | 2063 | 0.43 | 2.41 |  |
| I x T | 1761 | 0.45 | 2.78 |  |
| FR x P | 1686 | 0.43 | 2.24 |  |
| FR x M | 1610 | 0.42 | 2.39 |  |
| FR x T | 1492 | 0.45 | 2.70 |  |
| HR x P | 1013 | 0.32 | 1.99 |  |
| HR x M | 1124 | 0.34 | 1.99 |  |
| HR x T | 998 | 0.39 | 2.13 |  |

Different lowercase letters within the same column indicate significant difference at p< 0.05 according to Tukey's test. HSD: critical value for comparison. n.s.: not significant; significant at **p*<0.05; ***p* <0.01 and *** *p* < 0.001. I: irrigated. FR: fresh rainfed. HR hard rainfed; P: Pasto. M: Marisma. T: Titicaca
