# Supplementary material for: Assessment of the changes in seed yield and nutritional quality of quinoa grown under rainfed Mediterranean environments
Source: Front Plant Sci. 2023 Nov 3;14:1268014. doi: 10.3389/fpls.2023.1268014 (PMC10662129; doi:10.3389/fpls.2023.1268014)
Supplement: Supplementary file 4 [file Table_3.docx]

**Table S3.** Results of interactions between treatments for the proximate and mineral composition and saponin content of the seeds of three quinoa varieties (V) grown under three different water environmental conditions (WEC) during two consecutive years (Y).

| **Interaction** | **Humidity (g 100 g^-1^ fw)** | **Ash (g 100 g^-1^ fw)** | **Protein (g 100 g^-1^ fw)** | **Fat (g 100 g^-1^ fw)** | **Fibre (g 100 g^-1^ fw)** | **CH (g 100 g^-1^ fw)** | **Energy (Kcal 100 g^-1^ fw)** | **Saponin (g 100 g^-1^ fw)** | **P (%)** | **K (%)** | **Ca (%)** | **Mg (%)** | **Fe (ppm)** | **Na (ppm)** |
| --- | --- | --- | --- | --- | --- | --- | --- | --- | --- | --- | --- | --- | --- | --- |
| **Y x WEC** |  |  |  |  |  |  |  |  |  |  |  |  |  |  |
| 2019 x I | 9.5 b | 3.1 | 13.9 b | 5.2 | 7.9 | 60.7 | 360.4 | 0.65 c | 0.33 ab | 0.94 | 0.10 b | 0.21 | 67.6 | 237.5 a |
| 2019 x FR | 9.8 ab | 2.9 | 13.9 b | 5.7 | 9.0 | 58.8 | 359.6 | 1.56 a | 0.30 b | 1.11 | 0.16 a | 0.22 | 46.8 | 57.5 b |
| 2019 x HR | 9.5 b | 3.0 | 14.6 ab | 5.6 | 8.9 | 58.4 | 360.2 | 1.46 ab | 0.22 c | 1.06 | 0.11 ab | 0.16 | 42.4 | 61.5 b |
| 2020 x I | 10.6 a | 3.2 | 14.2 b | 5.2 | 10.5 | 56.3 | 349.9 | 1.15 b | 0.41 ab | 1.13 | 0.14 ab | 0.23 | 57.7 | 61.4 b |
| 2020 x FR | 10.0 ab | 3.3 | 13.6 b | 5.2 | 11.6 | 56.3 | 349.4 | 1.39 ab | 0.45 a | 1.22 | 0.12 ab | 0.24 | 66.5 | 49.1 b |
| 2020 x HR | 9.9 ab | 3.3 | 16.1 a | 5.3 | 10.9 | 54.6 | 351.6 | 1.46 ab | 0.38 ab | 1.19 | 0.13 ab | 0.20 | 54.2 | 56.6 b |
| **Y x V** |  |  |  |  |  |  |  |  |  |  |  |  |  |  |
| 2019 x P | 9.6 | 3.1 | 14.4 | 6.0 a | 9.0 bc | 57.9 ab | 360.9 ab | 1.20 | 0.32 ab | 1.13 abc | 0.13 | 0.22 | 62.0 | 127.2 |
| 2019 x M | 9.5 | 3.1 | 14.0 | 5.9 ab | 8.2 c | 59.4 a | 363.0 a | 1.14 | 0.29 bc | 1.08 bc | 0.13 | 0.20 | 48.9 | 106.8 |
| 2019 x T | 9.7 | 2.8 | 13.9 | 4.6 d | 8.2 c | 60.7 a | 356.2 bc | 1.40 | 0.24 c | 0.90 d | 0.11 | 0.18 | 45.9 | 122.5 |
| 2020 x P | 10.3 | 3.3 | 14.3 | 5.2 bcd | 11.7 ab | 55.2 bc | 348.6 cd | 1.40 | 0.40 ab | 1.19 ab | 0.12 | 0.22 | 61.2 | 58.9 |
| 2020 x M | 10.2 | 3.5 | 14.7 | 5.1 cd | 12.7 a | 53.9 c | 345.2 d | 1.39 | 0.44 a | 1.30 a | 0.13 | 0.23 | 62.0 | 54.9 |
| 2020 x T | 10.0 | 3.0 | 14.9 | 5.3 abc | 8.6 c | 58.0 ab | 357.0 ab | 1.21 | 0.40 ab | 1.04 cd | 0.13 | 0.21 | 55.1 | 53.2 |
| **WEC x V** |  |  |  |  |  |  |  |  |  |  |  |  |  |  |
| I x P | 10.3 | 3.3 | 13.3 | 5.5 | 9.1 ab | 58.3 ab | 354.6 ab | 1.01 | 0.40 | 1.13 | 0.15 | 0.23 | 68.5 | 165.9 |
| I x M | 9.7 | 3.2 | 14.7 | 5.2 | 8.5 ab | 58.7 ab | 357.2 ab | 0.93 | 0.38 | 1.10 | 0.11 | 0.23 | 60.2 | 131.7 |
| I x T | 10.1 | 2.8 | 14.1 | 4.9 | 9.6 ab | 58.4 ab | 353.6 ab | 0.75 | 0.34 | 0.88 | 0.11 | 0.20 | 59.1 | 150.6 |
| FR x P | 9.9 | 3.2 | 13.6 | 5.7 | 12.5 a | 55.1 b | 351.3 b | 1.53 | 0.39 | 1.19 | 0.12 | 0.24 | 56.5 | 58.4 |
| FR x M | 10.0 | 3.3 | 13.9 | 5.7 | 11.2 ab | 55.9 ab | 353.0 ab | 1.39 | 0.39 | 1.29 | 0.16 | 0.24 | 57.4 | 52.5 |
| FR x T | 9.8 | 2.9 | 13.6 | 4.9 | 7.2 b | 61.6 a | 359.1 a | 1.51 | 0.35 | 1.03 | 0.14 | 0.22 | 56.0 | 48.9 |
| HR x P | 9.6 | 3.1 | 16.1 | 5.6 | 9.4 ab | 56.2 ab | 358.4 ab | 1.37 | 0.30 | 1.18 | 0.11 | 0.19 | 59.7 | 54.8 |
| HR x M | 9.8 | 3.3 | 14.5 | 5.6 | 11.6 ab | 55.2 b | 352.1 ab | 1.47 | 0.32 | 1.19 | 0.13 | 0.19 | 48.9 | 58.3 |
| HR x T | 9.7 | 3.1 | 15.4 | 5.1 | 8.6 ab | 58.1 ab | 357.1 ab | 1.64 | 0.28 | 1.00 | 0.11 | 0.16 | 36.3 | 64.1 |
|  |  |  |  |  |  |  |  |  |  |  |  |  |  |  |

Different lowercase letters within the same column indicate significant difference at p< 0.05 according to Tukey's test. HSD: critical value for comparison. n.s.: not significant; significant at **p*<0.05; ***p* <0.01 and *** *p* < 0.001. I: irrigated. FR: fresh rainfed. HR: hard rainfed. P: Pasto. M: Marisma. T: Titicaca. Proximate composition and saponin content are expressed in fresh weight, while mineral composition is expressed in dry weight.
