# Supplementary material for: Assessment of the changes in seed yield and nutritional quality of quinoa grown under rainfed Mediterranean environments
Source: Front Plant Sci. 2023 Nov 3;14:1268014. doi: 10.3389/fpls.2023.1268014 (PMC10662129; doi:10.3389/fpls.2023.1268014)
Supplement: Supplementary file 5 [file Table_4.docx]

Table S4: Results of interactions between treatments for the main fatty acid content of the seeds of three quinoa varieties (V) grown under three different water environmental conditions (WEC) during two consecutive years (Y).

| Treatment | C16:0 | C18:1 | C18:2 | C18:3 | SFA | MUFA | PUFA | ω6/ω3 |
| --- | --- | --- | --- | --- | --- | --- | --- | --- |
| **Y x WEC** |  |  |  |  |  |  |  |  |
| 2019 x I | 9.71 | 20.5 | 59.9 | 6.0 | 10.8 | 23.1 | 66.1 | 10.1 |
| 2019 x FR | 9.59 | 20.1 | 61.0 | 5.9 | 10.7 | 22.2 | 67.0 | 10.5 |
| 2019 x HR | 9.62 | 20.1 | 61.8 | 5.3 | 10.6 | 22.2 | 67.2 | 11.8 |
| 2020 x I | 9.98 | 18.5 | 60.6 | 6.8 | 11.3 | 20.8 | 67.9 | 9.0 |
| 2020 x FR | 9.89 | 17.9 | 61.5 | 6.6 | 11.2 | 20.1 | 68.6 | 9.5 |
| 2020 x HR | 9.89 | 18.7 | 61.8 | 6.1 | 11.1 | 20.7 | 68.0 | 10.3 |
| **Y x V** |  |  |  |  |  |  |  |  |
| 2019 x P | 9.52 b | 20.7 a | 60.0 | 6.0 | 10.6 b | 23.2 a | 66.2 c | 10.1 |
| 2019 x M | 9.69 b | 20.1 ab | 60.7 | 5.9 | 10.8 b | 22.5 ab | 66.7 bc | 10.4 |
| 2019 x T | 9.72 ab | 19.9 ab | 62.0 | 5.3 | 10.7 b | 21.8 bc | 67.4 ab | 11.8 |
| 2020 x P | 10.03 ab | 18.2 cd | 60.8 | 6.9 | 11.4 a | 20.4 c | 68.1 ab | 8.8 |
| 2020 x M | 10.05 a | 17.7 d | 61.5 | 6.7 | 11.4 a | 20.0 c | 68.6 a | 9.3 |
| 2020 x T | 9.67 b | 19.1 bc | 61.6 | 5.9 | 10.9 b | 21.2 bc | 67.8 ab | 10.6 |
| **WEC x V** |  |  |  |  |  |  |  |  |
| I x P | 9.82 | 19.8 | 59.4 | 6.6 | 11.1 | 22.6 | 66.3 | 9.0 |
| I x M | 10.05 | 19.9 | 60.2 | 6.7 | 11.3 | 21.4 | 67.2 | 9.1 |
| I x T | 9.66 | 18.9 | 61.2 | 5.9 | 10.7 | 21.9 | 67.3 | 10.4 |
| FR x P | 9.76 | 19.1 | 60.7 | 6.6 | 11.0 | 21.3 | 67.7 | 9.3 |
| FR x M | 9.85 | 18.8 | 61.1 | 6.4 | 11.1 | 21.0 | 67.9 | 9.6 |
| FR x T | 9.60 | 19.1 | 61.9 | 5.7 | 10.8 | 21.2 | 67.9 | 11.0 |
| HR x P | 9.73 | 19.5 | 61.2 | 6.2 | 10.9 | 21.6 | 67.5 | 10.1 |
| HR x M | 9.72 | 19.2 | 61.9 | 5.8 | 10.9 | 21.3 | 67.8 | 10.8 |
| HR x T | 9.81 | 19.6 | 62.3 | 5.1 | 10.9 | 21.5 | 67.6 | 12.2 |

Different lowercase letters within the same column indicate significant difference at p< 0.05 according to Tukey's test. HSD: critical value for comparison. n.s.: not significant; significant at **p*<0.05; ***p* <0.01 and *** *p* < 0.001. I: irrigated. FR: fresh rainfed. HR: hard rainfed. P: Pasto. M: Marisma. T: Titicaca
