# Supplementary material for: Assessment of the changes in seed yield and nutritional quality of quinoa grown under rainfed Mediterranean environments
Source: Front Plant Sci. 2023 Nov 3;14:1268014. doi: 10.3389/fpls.2023.1268014 (PMC10662129; doi:10.3389/fpls.2023.1268014)
Supplement: Supplementary file 6 [file Table_5.docx]

**Table S5.** Results of interactions between treatments for the minor fatty acids contents in seeds harvested from three quinoa varieties (Pasto, Marisma, and Titicaca) grown under three different environmental conditions (I, FR, HR) during two consecutive years (2019, 2020).

| Treatment | C14:0 | C15:0 | C16:1 | C17:0 | C17:1 | C18:0 | C20:0 | C20:1 | C20:2 | C22:0 | C22:1 |
| --- | --- | --- | --- | --- | --- | --- | --- | --- | --- | --- | --- |
| **Y x WEC** |  |  |  |  |  |  |  |  |  |  |  |
| 2019 x I | 0.267 ab | ^1^ | 0.152 | 0.049 | 0.071 bc | 0.387 | 0.280 b | 1.886 | 0.177 b | 0.132 bc | 0.517 |
| 2019 x FR | 0.279 a |  | 0.137 | 0.045 | 0.067 cd | 0.419 | 0.288 ab | 1.399 | 0.160 b | 0.107 c | 0.495 |
| 2019 x HR | 0.273 ab |  | 0.176 | 0.041 | 0.062 cd | 0.508 | 0.233 c | 1.317 | 0.147 b | 0.108 c | 0.468 |
| 2020 x I | 0.239 cd |  | 0.058 | 0.048 | 0.097 ab | 0.541 | 0.310 ab | 1.503 | 0.448 a | 0.167 ab | 0.590 |
| 2020 x FR | 0.237 d |  | 0.048 | 0.049 | 0.111 a | 0.546 | 0.283 b | 1.497 | 0.512 a | 0.193 a | 0.592 |
| 2020 x HR | 0.258 bc |  | 0.044 | 0.041 | 0.039 d | 0.508 | 0.326 a | 1.370 | 0.146 b | 0.129 bc | 0.567 |
| **Y x V** |  |  |  |  |  |  |  |  |  |  |  |
| 2019 x P | 0.270 | ^1^ | 0.150 | 0.041 | 0.068 ab | 0.392 | 0.278 | 1.738 | 0.170 | 0.131 | 0.518 |
| 2019 x M | 0.272 |  | 0.167 | 0.045 | 0.085 ab | 0.369 | 0.267 | 1.576 | 0.156 | 0.136 | 0.523 |
| 2019 x T | 0.277 |  | 0.148 | 0.049 | 0.046 b | 0.363 | 0.255 | 1.289 | 0.157 | 0.080 | 0.436 |
| 2020 x P | 0.240 |  | 0.059 | 0.042 | 0.083 ab | 0.550 | 0.310 | 1.482 | 0.353 | 0.180 | 0.592 |
| 2020 x M | 0.250 |  | 0.058 | 0.051 | 0.091 a | 0.540 | 0.306 | 1.450 | 0.416 | 0.186 | 0.619 |
| 2020 x T | 0.243 |  | 0.033 | 0.044 | 0.072 ab | 0.504 | 0.303 | 1.438 | 0.337 | 0.123 | 0.538 |
| **WEC x V** |  |  |  |  |  |  |  |  |  |  |  |
| I x P | 0.251 | 0.043 | 0.116 | 0.047 | 0.091 abc | 0.511 | 0.311 | 1.986 | 0.322 | 0.175 a | 0.595 |
| I x M | 0.256 | 0.043 | 0.116 | 0.052 | 0.107 a | 0.473 | 0.290 | 1.733 | 0.410 | 0.180 a | 0.565 |
| I x T | 0.253 | 0.053 | 0.080 | 0.047 | 0.053 cd | 0.409 | 0.285 | 1.365 | 0.205 | 0.093 b | 0.496 |
| FR x P | 0.254 | 0.043 | 0.090 | 0.042 | 0.087 abcd | 0.471 | 0.278 | 1.462 | 0.321 | 0.157 ab | 0.542 |
| FR x M | 0.254 | 0.040 | 0.096 | 0.049 | 0.100 ab | 0.480 | 0.289 | 1.350 | 0.304 | 0.167 a | 0.592 |
| FR x T | 0.264 | 0.037 | 0.091 | 0.050 | 0.080 abcd | 0.497 | 0.290 | 1.298 | 0.383 | 0.127 ab | 0.497 |
| HR x P | 0.259 | 0.090 | 0.105 | 0.036 | 0.049 cd | 0.431 | 0.294 | 1.382 | 0.142 | 0.135 ab | 0.529 |
| HR x M | 0.273 | 0.080 | 0.125 | 0.044 | 0.058 bcd | 0.411 | 0.280 | 1.350 | 0.143 | 0.135 ab | 0.556 |
| HR x T | 0.263 | 0.067 | 0.100 | 0.043 | 0.044 d | 0.396 | 0.263 | 1.365 | 0.154 | 0.086 b | 0.467 |

^1^not detected in 2019. Myristic acid (C14:0); pentadecanoic acid (C15:0) palmitoleic acid (C16:1); margaric acid (C17:0); margaroleic acid (C17:1); stearic acid (C18:0); arachidic acid (C20:0); gadoleic acid (C20:1); eicosadienoic acid (C20:2); behenic acid (C22:0); and erucic acid (C22:1). Different lowercase letters within the same column indicate significant difference at p< 0.05 according to Tukey's test. HSD: critical value for comparison. n.s.: not significant; significant at **p*<0.05; ***p* <0.01 and *** *p* < 0.001. I: irrigated. FR: fresh rainfed. HR: hard rainfed. P: Pasto; M: Marisma; T: Titicaca.
