# Supplementary material for: Assessment of the changes in seed yield and nutritional quality of quinoa grown under rainfed Mediterranean environments
Source: Front Plant Sci. 2023 Nov 3;14:1268014. doi: 10.3389/fpls.2023.1268014 (PMC10662129; doi:10.3389/fpls.2023.1268014)
Supplement: Supplementary file 7 [file Table_6.docx]

| **Chemical Shift (ppm)** | **Metabolites** |  | **Chemical Shift (ppm)** | **Metabolites** |  | **Chemical Shift (ppm)** | **Metabolites** |  | **Chemical Shift (ppm)** | **Metabolites** |
| --- | --- | --- | --- | --- | --- | --- | --- | --- | --- | --- |
| **Organic Acids** | |  | **Aminoacids** | |  | **Sugars** | |  | **Others** | |
| 8.43 (s) | Formic acid |  | 2.35 (dt) | Glutamate |  | 5.22 (d) | Glucose |  | 3.19 (s) | Choline |
| 4.31 (dd) | Malic Acid |  | 1.46 (d) | Alanine |  | 4.01 (m) | Fructose |  | 9.11 (s) | Trigonelline |
| 2.37 (s) | Succinic Acid |  | 1.32 (d) | Threonine |  | 5.40 (d) | Sucrose |  | 3.13 (t) | Ethanolamine |
| 2.69 (d) | Citric Acid |  | 2,3 (t) | 4-Aminobutyrate |  | 3,26 (t) | *myo*-Inositol |  | 3.27 y 3.91 (s) | Glycine-Betaine |
| 6,53 (s) | Fumaric Acid |  | 1,03 (d) | Isoleucine |  | 5.18 (d) | Trehalose |  | 3,8 y 4,6 (m) | Glutathione |
| 4,51 (d) | Ascorbic acid |  | 0,98 (t) | Leucine |  | 5.42 (d) | Maltose |  |  |  |
| 4,1 (d) | Glucuronic acid |  | 1.02 (d) | Valine |  |  |  |  |  |  |
| 1,3 (d) | Lactic acid |  | 3,6 (s) | Glycine |  |  |  |  |  |  |
|  |  |  | 2.94 (dd) | Asparagine |  |  |  |  |  |  |
|  |  |  | 2.81 (dd) | Aspartate |  |  |  |  |  |  |
|  |  |  | 7.41 (m) | Phenylalanine |  |  |  |  |  |  |
|  |  |  | 6.88 (d) | Tyrosine |  |  |  |  |  |  |
|  |  |  | 2,0 (m) | Proline |  |  |  |  |  |  |
|  |  |  | 4.16 (dd) | Pyroglutamate |  |  |  |  |  |  |
|  |  |  | 2.1 (m) | Glutamine |  |  |  |  |  |  |

**Table S6.** Chemical shift (ppm) of metabolites

**s**, singlet; **d**, doublet; **t**, triplet; **dd**, double of doublets; **m**, multiplet
