# Supplementary material for: Assessment of the changes in seed yield and nutritional quality of quinoa grown under rainfed Mediterranean environments
Source: Front Plant Sci. 2023 Nov 3;14:1268014. doi: 10.3389/fpls.2023.1268014 (PMC10662129; doi:10.3389/fpls.2023.1268014)
Supplement: Supplementary file 8 [file Table_7.docx]

**Table S7**. Statistical analysis for metabolites determined in seeds from Pasto, Marisma, and Titicaca , cultivated under irrigated (I), fresh rainfed (FR), and hard rainfed (HR) field conditions during two consecutive years (2019 and 2020). Values are expressed as µmol per gram of seed extract (dw = dry weight). Values represent the mean of three biological replicates followed by a letter, indicating statistically different groups calculated by one-way ANOVA followed by Tukey’s multiple comparisons (*p ≤ 0.05*). When analysing factor interaction *, **, *** indicate statistically significant differences in mean values with a significance level *0.01 ˂ p ≤ 0.05*, *0.001 ˂ p ≤ 0.01,* and *p ≤ 0.001*, respectively; ns = no statistically significant differences.

| Sugars | | | | | |
| --- | --- | --- | --- | --- | --- |
|  | | | | | |
| Factor | | **Glucose**  (µmol/g dW) | **Maltose**  (µmol/g dw) | **Myo**-**Inositol**  (µmol/g dw) | **Sucrose**  (µmol/g dw) |
| Year (Y) | **2019** | 21.89 a | 7.69 a | 5.18 a | 94.16 b |
|  | **2020** | 15.25 a | 8.65 a | 4.75 a | 104.44 a |
|  | | | | | |
| Water environmental  conditions (WEC) | **I** | 30.38 a | 7.89 a | 5.37 a | 92.97 b |
|  | **FR** | 15.31 b | 8.40 a | 5.12 a | 99.88 ab |
|  | **HR** | 10.02 b | 8.22 a | 4.40 a | 105.06 a |
|  | | | | | |
| Variety (V) | **Pasto** | 23.95 a | 8.46 a | 5.81 a | 103.84 a |
|  | **Marisma** | 19.85 ab | 8.26 a | 4.75 b | 98.85 a |
|  | **Titicaca** | 11.90 b | 7.79 a | 4.33 b | 95.21 a |
| Factor interaction | | | | | |
| Y | | ns | ns | ns | ** |
| WEC | | *** | ns | ns | ns |
| V | | ** | ns | ** | ns |
| Y x WEC | | *** | ns | ** | ** |
| Y x V | | * | ns | ns | ** |
| WEC x V | | *** | ns | *** | ns |
| Y x WEC x V | | *** | ns | *** | ns |

| Free amino acids | | | | | | | | | | |
| --- | --- | --- | --- | --- | --- | --- | --- | --- | --- | --- |
|  | | | | | | | | | | |
| Factor | | **Alanine**  (µmol/g DW) | **Aspartate**  (µmol/g DW) | **GABA**  (µmol/g DW) | **Glutamate**  (µmol/g DW) | **Glycine**  (µmol/g DW) | **Phenylalanine**  (µmol/g DW) | **Threonine**  (µmol/g DW) | **Tyrosine**  (µmol/g DW) | **Valine**  (µmol/g DW) |
| Year (Y) | **2019** | 2.03 b | 2.79 b | 0.16 b | 5.45 b | 7.77 a | 0.00 b | 0.00 b | 0.25 b | 0.88 b |
|  | **2020** | 2.79 a | 3.63 a | 0.42 a | 6.42 a | 4.66 b | 0.34 a | 0.43 a | 0.56 a | 1.15 a |
|  | | | | | | | | | |  |
| Water environmental conditions (WEC)) | **I** | 2.82 a | 3.32 ab | 0.52 a | 5.82 a | 7.53 a | 0.23 ab | 0.22 a | 0.42 a | 1.14 a |
|  | **FR** | 2.66 a | 3.63 a | 0.32 b | 6.09 a | 5.40 b | 0.28 a | 0.30 a | 0.53 a | 1.15 a |
|  | **HR** | 1.76 b | 2.68 b | 0.00 c | 5.90 a | 5.73 ab | 0.00 b | 0.12 a | 0.27 a | 0.75 b |
|  | | | | | | | | | |  |
| Variety (V) | **Pasto** | 2.61 a | 3.66 a | 0.33 a | 5.96 a | 6.57 a | 0.28 a | 0.16 a | 0.42 a | 1.16 a |
|  | **Marisma** | 2.57 a | 3.42 a | 0.31 a | 5.93 a | 6.39 a | 0.23 ab | 0.31 a | 0.35 a | 1.11 a |
|  | **Titicaca** | 2.06 a | 2.56 b | 0.21 a | 5.92 a | 5.70 a | 0.00b | 0.17 a | 0.45 a | 0.77 b |
| Factor interaction | | | | | | | | | |  |
| Y | | *** | ** | *** | *** | *** | *** | *** | ** | * |
| WEC | | *** | * | *** | ns | * | * | ns | ns | ** |
| V | | ns | ** | ns | ns | ns | * | ns | ns | ** |
| Y x WEC | | *** | *** | *** | *** | *** | *** | *** | ** | *** |
| Y x V | | *** | *** | * | *** | *** | *** | *** | ns | ** |
| WEC x V | | *** | ** | *** | ns | ns | ** | ns | ns | ** |
| Y x WEC x V | | *** | *** | *** | ** | *** | *** | *** | ns | *** |

| Organic acids | | | | | | | | |
| --- | --- | --- | --- | --- | --- | --- | --- | --- |
| Factor | | **Acetate**  (µmol/g DW) | **Citrate**  (µmol/g DW) | **Formate**  (µmol/g DW) | **Glucuronate**  (µmol/g DW) | **Lactate**  (µmol/g DW) | **Malate**  (µmol/g DW) | **Succinate**  (µmol/g DW) |
| Year (Y) | **2019** | 1.48 a | 5.06 b | 1.16 a | 0.073 a | 0.000 b | 4.61 b | 0.83 a |
|  | **2020** | 1.22 b | 6.23 a | 0.77 b | 0.000 b | 0.475 a | 5.65 a | 0.74 a |
|  | | | | | | | | |
| Water Environmental conditions (WEC) | **I** | 1.44 a | 5.68 a | 1.01 a | 0.109a | 0.328 a | 5.64 a | 0.78 a |
|  | **FR** | 1.51 a | 5.49 a | 0.95 a | 0.000 b | 0.251 a | 5.05 a | 0.75 a |
|  | **HR** | 1.10 b | 5.77 a | 0.93 a | 0.000 b | 0.134 a | 4.70 a | 0.83 a |
|  | | | | | | | | |
| Variety (V) | **Pasto** | 1.30 a | 5.79 a | 1.06 a | 0.061 a | 0.178 a | 6.20 a | 0.94 a |
|  | **Marisma** | 1.44 a | 5.52 a | 1.01 ab | 0.029 a | 0.320 a | 5.52 a | 0.86 a |
|  | **Titicaca** | 1.22 a | 5.63 a | 0.82 b | 0.020 a | 0.214 a | 3.67 b | 0.56 b |
| Factor interaction | | | | | | | | |
| Y | | ** | *** | *** | ** | *** | * | ns |
| WEC | | *** | ns | ns | *** | ns | ns | ns |
| V | | ns | ns | * | ns | ns | *** | *** |
| Y x WEC | | *** | *** | *** | *** | *** | * | ns |
| Y x V | | * | * | *** | * | *** | *** | *** |
| WEC x V | | *** | ns | ns | ** | ns | *** | *** |
| Y x WEC x V | | *** | *** | *** | *** | *** | *** | *** |

| Secondary metabolites | | | | | |
| --- | --- | --- | --- | --- | --- |
| Factor | | Betaine  (µmol/g DW) | Choline  (µmol/g DW) | | Trigonelline  (µmol/g DW) |
| Year (Y) | 2019 | 66.51 a | 3.42 b | | 0.62 b |
|  | 2020 | 68.49 a | 5.93 a | | 0.81 a |
|  | | | | | |
| Water environmental conditions (WEC) | I | 55.22 c | 3.89 a | | 0.66 a |
|  | FR | 67.63 b | 4.55 a | | 0.77 a |
|  | HR | 79.65 a | 5.59 a | | 0.72 a |
|  | | | | | |
| Variety (V) | Pasto | | 65.51 a | 4.58 a | 0.71 a |
|  | Marisma | | 67.71 a | 5.12 a | 0.71 a |
|  | Titicaca | | 69.28 a | 4.33 a | 0.73 a |
| Factor interaction | | | | | |
| Y | | ns | *** | | *** |
| WEC | | *** | ns | | ns |
| V | | ns | ns | | ns |
| Y x WEC | | *** | *** | | *** |
| Y x V | | ns | *** | | *** |
| WEC x V | | *** | ns | | ns |
| Y x WEC x V | | *** | ** | | * |
